# Molecular mechanism of siderophore regulation by the *Pseudomonas aeruginosa* BfmRS two-component system in response to osmotic stress

**DOI:** 10.1038/s42003-024-05995-z

**Published:** 2024-03-09

**Authors:** Yingjie Song, Xiyu Wu, Ze Li, Qin qin Ma, Rui Bao

**Affiliations:** 1 College of Life Science, Sichuan Normal University, Chengdu, 610101 China; 2 Advanced Mass Spectrometry Center, Research Core Facility, Frontiers Science Center for Disease-related Molecular Network, West China Hospital, Sichuan University, Chengdu, 610213 China; 3 Center of Infectious Diseases, Division of Infectious Diseases in State Key Laboratory of Biotherapy, West China Hospital, Sichuan University, Chengdu, 610041 China

**Keywords:** Pathogens, Bacterial pathogenesis

## Abstract

*Pseudomonas aeruginosa*, a common nosocomial pathogen, relies on siderophores to acquire iron, crucial for its survival in various environments and during host infections. However, understanding the molecular mechanisms of siderophore regulation remains incomplete. In this study, we found that the BfmRS two-component system, previously associated with biofilm formation and quorum sensing, is essential for siderophore regulation under high osmolality stress. Activated BfmR directly bound to the promoter regions of *pvd, fpv*, and *femARI* gene clusters, thereby activating their transcription and promoting siderophore production. Subsequent proteomic and phenotypic analyses confirmed that deletion of BfmRS reduces siderophore-related proteins and impairs bacterial survival in iron-deficient conditions. Furthermore, phylogenetic analysis demonstrated the high conservation of the BfmRS system across *Pseudomonas* species, functional evidences also indicated that BfmR homologues from *Pseudomonas putida* KT2440 and *Pseudomonas* sp. MRSN12121 could bind to the promoter regions of key siderophore genes and osmolality-mediated increases in siderophore production were observed. This work illuminates a novel signaling pathway for siderophore regulation and enhances our understanding of siderophore-mediated bacterial interactions and community establishment.

## Abstract (web summary)

A comprehensive study reveals that the two-component system BfmRS senses high osmotic stress and transcriptionally activates the expression of siderophore genes, which is essential for environmental adaption of *P. aeruginosa*.

## Introduction

P*seudomonas aeruginosa*, a notorious opportunistic human pathogen, poses significant challenges due to its capacity to induce acute and chronic infections, particularly among immunocompromised patients^1–3^. It is the primary culprit behind chronic pulmonary infections and subsequent mortality in cystic fibrosis (CF) patients worldwide^4,5^. To establish itself as the dominant organism in the CF airway, *P. aeruginosa* deploys an array of well-characterized virulence factors, including elastase, pyocyanin, and others^6^. These effectors not only undermine the host immune system but also confer an ecological advantage. However, like many pathogenic bacteria, the survival and proliferation of *P. aeruginosa*, both within and outside the host, rely on the availability of iron^7,8^. Iron is indispensable for fundamental cellular processes in microorganisms, yet its bioavailability is inherently limited due to its oxidized state (Fe^3+^), which is largely insoluble under natural conditions^9^. To circumvent iron scarcity, bacteria, including *P. aeruginosa*, have evolved the capacity to synthesize and secrete siderophores^7,10,11^. Siderophores are chemically diverse secondary metabolites that form soluble complexes with free Fe^3+^ in the environment. Subsequently, these complexes are actively acquired by the bacteria through specific receptors. Consequently, siderophores play a critical role in iron acquisition throughout the lifecycle of *P. aeruginosa*^7,9^. Given the clinical significance of *P. aeruginosa* and the challenges posed by its multidrug resistance, understanding its iron metabolism, particularly the role of siderophores, becomes crucial.

Aside from its other unique attributes, *P. aeruginosa* exhibits a remarkable proficiency in synthesizing two distinct siderophores: pyoverdine (PVDI) and pyochelin (PCH)^7,8,12^. This capability sets it apart from other bacteria, underscoring the complexity and intricacy of its siderophore synthesis pathways and mechanisms. PVDI is synthesized by non-ribosomal peptide synthetases associated with the *pvdA* cluster within the bacterial cytoplasm, after which it is secreted into the extracellular environment^10,12^. Once PVDI chelates ferric iron, it is transported back into the *P. aeruginosa* periplasm through the FpvAI and FpvB transporters. Subsequently, the iron is released from PVDI and undergoes further reduction through a cascade of proteins encoded by the *fpvGHJKCDEF* gene cluster^13^. Similarly, PCH is synthesized and transported by proteins encoded by the *pchABCDEF* cluster^11^. Apart from its role in iron acquisition, it is worth noting that PCH also serves as a signaling molecule, capable of eliciting the expression of virulence factors in *P. aeruginosa*, thus influencing its pathogenicity^9^. Research has indicated that PCH production is associated with biofilm formation, while PVDI exhibits protective effects against reactive oxygen species (ROS) induced by external conditions and simultaneously exerts a toxic influence on host cells^10^. In light of these multifaceted functions, enhancing our understanding of the evolutionary and adaptive aspects of siderophore regulation in *P. aeruginosa* becomes essential. Such insights hold the potential to inform the development of more effective strategies for the clinical treatment of *P. aeruginosa* infections.

The regulation of siderophore biosynthesis and uptake is generally conserved among *Pseudomonas* species, typically involving the presence of the conserved master regulator Fur-like protein^10,11^. However, in certain bacterial strains, iron-acquisition systems exhibit a more intricate regulatory network, with additional regulatory elements contributing to their control^14,15^. Emerging evidence suggests that two-component systems (TCSs) play a pivotal role in governing iron uptake processes^1,16^. Canonical TCSs consist of a sensor histidine kinase (HK) and a cognate response regulator (RR), functioning as common signal transduction cascades in bacteria to perceive environmental cues, including ions, antibiotics, compounds, and peptides^17^. This sensory input ultimately leads to the transcriptional activation of relevant target genes. *P. aeruginosa* boasts an extensive repertoire of TCSs and has been shown to employ several of them in the regulation of iron metabolism^16^. For example, GacAS controls quorum sensing (QS), virulence factors, biofilm formation, antibiotic resistance, swarming, and motility in *P. aeruginosa*, although its direct impact on siderophore production is limited^18^. Conversely, PirRS governs the transcription of a TonB-dependent transporter, PirA, which plays a role in the uptake of iron chelated by mono-catechol^19^. Meanwhile, PfeRS is responsible for the uptake of heterologous siderophores by sensing enterobactin^20^. These findings underscore the significance of TCSs as crucial and adaptable mechanisms that enable *P. aeruginosa* to sense and respond to environmental changes, thereby modulating its iron metabolism. On the other hand, evidence has demonstrated that the production of siderophores is induced in *P. aeruginosa* and *Pseudomonas putida* under high osmolarity conditions, which may be related to adaptations to the host environments^21^. Although little is known about this, the OmpR/EnvZ TCS in *Escherichia coli* is able to regulate iron acquisition by sensing increasing osmolytes, highlighting the unusual regulation of osmotic stress on iron uptake process via TCSs^22^. Nevertheless, the precise role of TCSs in the intricate control of essential iron metabolism in *P. aeruginosa* remains incompletely understood and merits further investigation.

The BfmRS (biofilm maturation) TCS has previously been reported to be essential for biofilm maturation and the activation of *rhl* QS system in *P. aeruginosa*^23,24^. This regulation occurs through the control of *phdA* and *rhlR* expression, respectively. Further studies emphasized that mutation-induced activation of BfmRS in *P. aeruginosa* isolates could potentially contribute to host adaptation during infections^25,26^. These observations underscore the significance of the BfmRS system, yet further exploration of its complex regulatory activities within *P. aeruginosa* is ongoing. Here, we demonstrate that the BfmRS system shares homology with the OmpR/EnvZ family, a TCS involved in bacterial osmoadaptation. Furthermore, we reveal its contribution to siderophore production in response to changes in medium osmolarity. Our study indicates that BfmR significantly enhances siderophore synthesis by directly binding to the promoters of the *pvd*, *fpv*, and *femARI* clusters under conditions of elevated osmolarity. Subsequent proteomics analysis unveiled that the deletion of *bfmRS* resulted in reduced expression levels of proteins associated with siderophores. This reduction led to a substantial decrease in siderophore production and impaired bacterial survival in a mouse infection model. Additionally, phylogenetic analysis confirmed the widespread conservation of the BfmRS system across different *Pseudomonas* species. In *P. putida* KT2440 and *Pseudomonas* sp. MRSN12121, BfmR homologs could also bind to the promoter regions of siderophore genes, and similar osmolality-mediated increases in siderophore production were observed. In summary, our findings shed light on a novel mechanism by which the BfmRS system enables *Pseudomonas* species to sense and respond to the presence of hosts or changes in various ecological niches. This specific regulation holds promise for the development of new strategies targeting iron metabolism to combat *P. aeruginosa* infections.

## Results

### Revealing the osmotic sensing capabilities and siderophore regulation by *P. aeruginosa* BfmRS

**Fig. 1:**
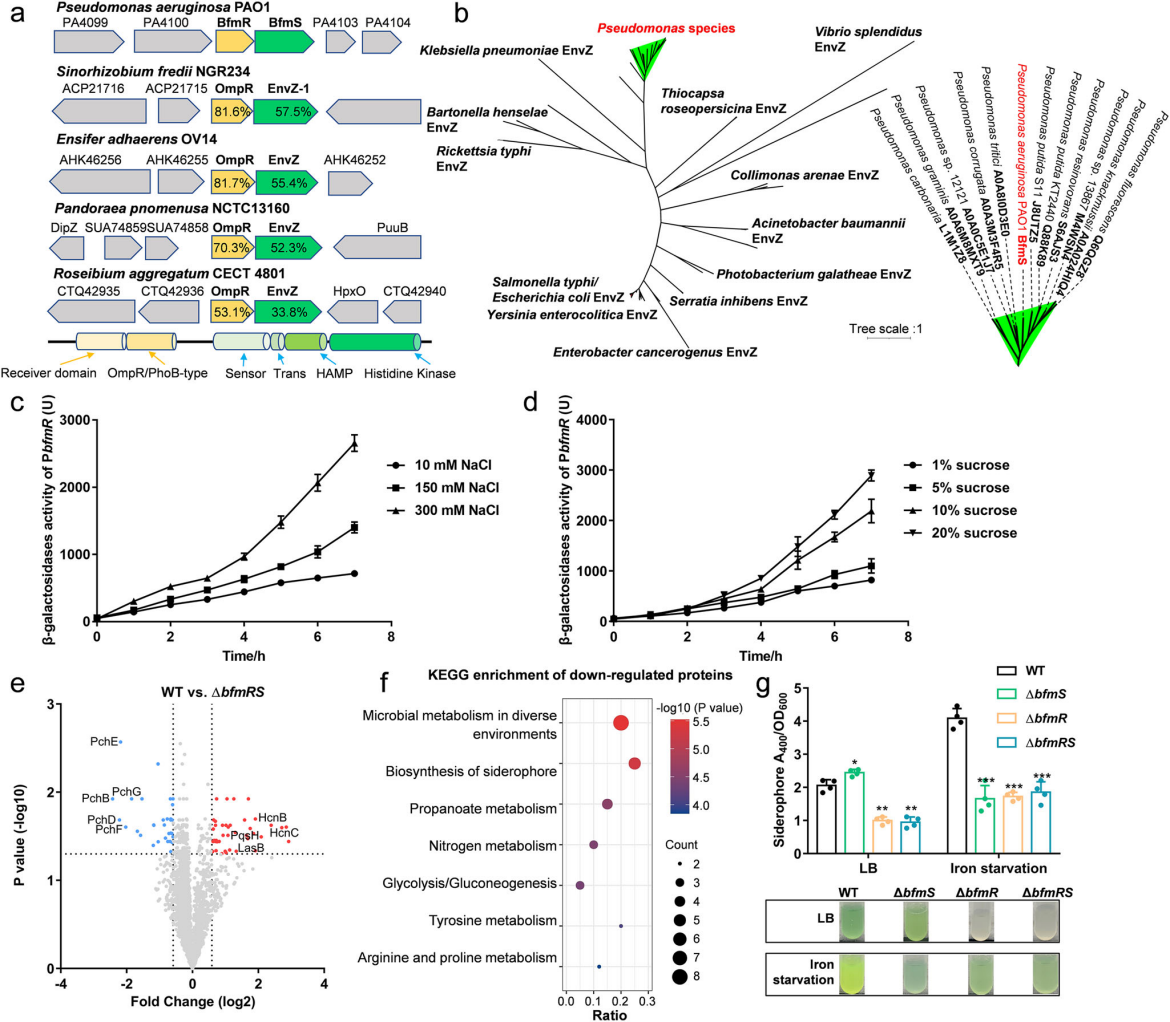
BfmR/BfmS is a conserved TCS of OmpR/EnvZ family across *Pseudomonas* species. **a** Schematic representation of the operon and surrounding genomic loci of the BfmRS system and homologs in bacteria. **b** Phylogeny of BfmS homologs from *Pseudomonas* species and other identified EnvZ proteins in bacteria. Detailed information on BfmS homologs is listed in right panel. **c**, **d** Construction of β-galactosidase reporter system to determine the transcriptional activities of *bfmRS* operon in *P. aeruginosa* under diverse osmotic stresses. **e** Volcano plot displaying the proteomic profiles of WT and Δ*bfmRS* strains. The significantly up and downregulated proteins are labeled with red and cyan, respectively. The downregulated proteins were also categorized by KEGG enrichment functional category (**f**). **g** Siderophore production of WT, Δ*bfmS*, Δ*bfmR,* and Δ*bfmRS* strains in normal condition or iron starvation condition. The experiments were performed four times, and the data were presented as the average ± SD (error bar). *P < 0.05; **P < 0.01; ***P < 0.001.

The *bfmRS*-like operons are prevalent in the genomes of many Gram-negative bacteria^27,28^. BfmRS TCS has been implicated in controlling stress response and even light signal transduction^29^. However, the specific signals that directly stimulate BfmS, the sensor component of this system, remained elusive. In *P. aeruginosa* PAO1, *pa4102* encoded BfmS has been proven to negatively control the activity of BfmR, which is encoded by *pa4101* and responsible for biofilm maturation and the *rhl* QS system^24^. Through homology-based BLAST searches and comparisons, it can be observed that *P. aeruginosa* BfmRS is widespread across diverse bacterial taxa and shares high similarity to the putative OmpR/EnvZ pairs from *Sinorhizobium fredii* NGR234, *Ensifer adhaerens* OV14, and *Pandoraea pnomenusa* NCTC13160 (Fig. 1a, Supplementary Fig. 1a). OmpR/EnvZ systems were identified as regulators of outer membrane protein expression in response to changes in medium osmolality^30^. The distinguishing feature of these systems lies in the osmosensing core, which has been found to be located not in the N-terminal domain, but in the C-terminal cytoplasmic domain^30^. Alignment analysis revealed that BfmS has a highly conserved Histidine-containing osmosensor region as other EnvZ proteins (Supplementary Fig. 1b), suggesting that BfmS possesses structural elements with the potential to sense osmotic changes. To further investigate the relationship, we conducted a phylogenetic analysis using 43 known or putative EnvZ proteins from pathogenic bacteria and 12 BfmS orthologs from *Pseudomonas* species (Fig. 1b). Despite their relatively low sequence identities (16.68–30.72%), the BfmS homologs clustered into a distinct subfamily (Fig. 1b).

We then investigated the expression of *bfmRS* in *P. aeruginosa* PAO1 using β-galactosidase reporter gene constructs under varying osmolarities. As shown in Fig. 1c, d, the transcriptional activities of *bfmRS* promoter (P_*bfmR*_) were increased 3.52- to 3.71-fold under high osmotic stresses induced by either NaCl or sucrose, as compared to conditions of low osmolality. This indicates that the expression of the *bfmRS* operon is intrinsically regulated in response to high osmotic pressure.

To explore the signaling pathways associated with the BfmRS system, we performed a global proteomic analysis by comparing *P. aeruginosa* Δ*bfmRS* with the wild-type (WT) *P. aeruginosa* PAO1. Among the 3408 surveyed proteins, 73 differentially expressed proteins (DEPs) were identified in Δ*bfmRS* compared to the WT strain (Fig. 1e, Supplementary Data 1). Potential functions of these DEPs according to GO enrichment and KEGG pathway associations were further analyzed (Fig. 1f). In alignment with prior reports, approximately 25% of the proteins with elevated expression levels were linked to biofilm formation and metabolic pathways. Additionally, several ABC transporters and proteins associated with QS exhibited significant increases^31^. Specifically, proteins involved in siderophore biosynthesis, including PCH synthase PchB, PchD, PchE, PchF, and PchG, displayed reduced expression compared to the WT strain (Fig. 1e). This observation suggests an association between the BfmRS system and siderophore metabolism. Based on that, we proceeded to investigate siderophore production, which was found to be markedly decreased in Δ*bfmR* and Δ*bfmRS* strains when compared to the WT strain both in LB medium and iron deficiency medium (Fig. 1g). Meanwhile, Δ*bfmS* showed significant decrease of siderophore production only in iron starvation medium. This is consistent with previous study that BfmS serves as a net kinase (kinase activity > phosphatase activity) or a net phosphatase (phosphatase activity >kinase activity) to modulate the status of BfmR in diverse conditions^26^. Thus, BfmS might decrease the activities of BfmR in common LB medium and enhance the activation of BfmR under iron starvation conditions. Moreover, complementation of each gene in these deletion mutants restored the siderophore production (Supplementary Fig. 2). These results collectively underscore the positive role of BfmRS in siderophore production.

### The significance of the BfmRS system in osmotic stress-induced siderophore metabolism

**Fig. 2:**
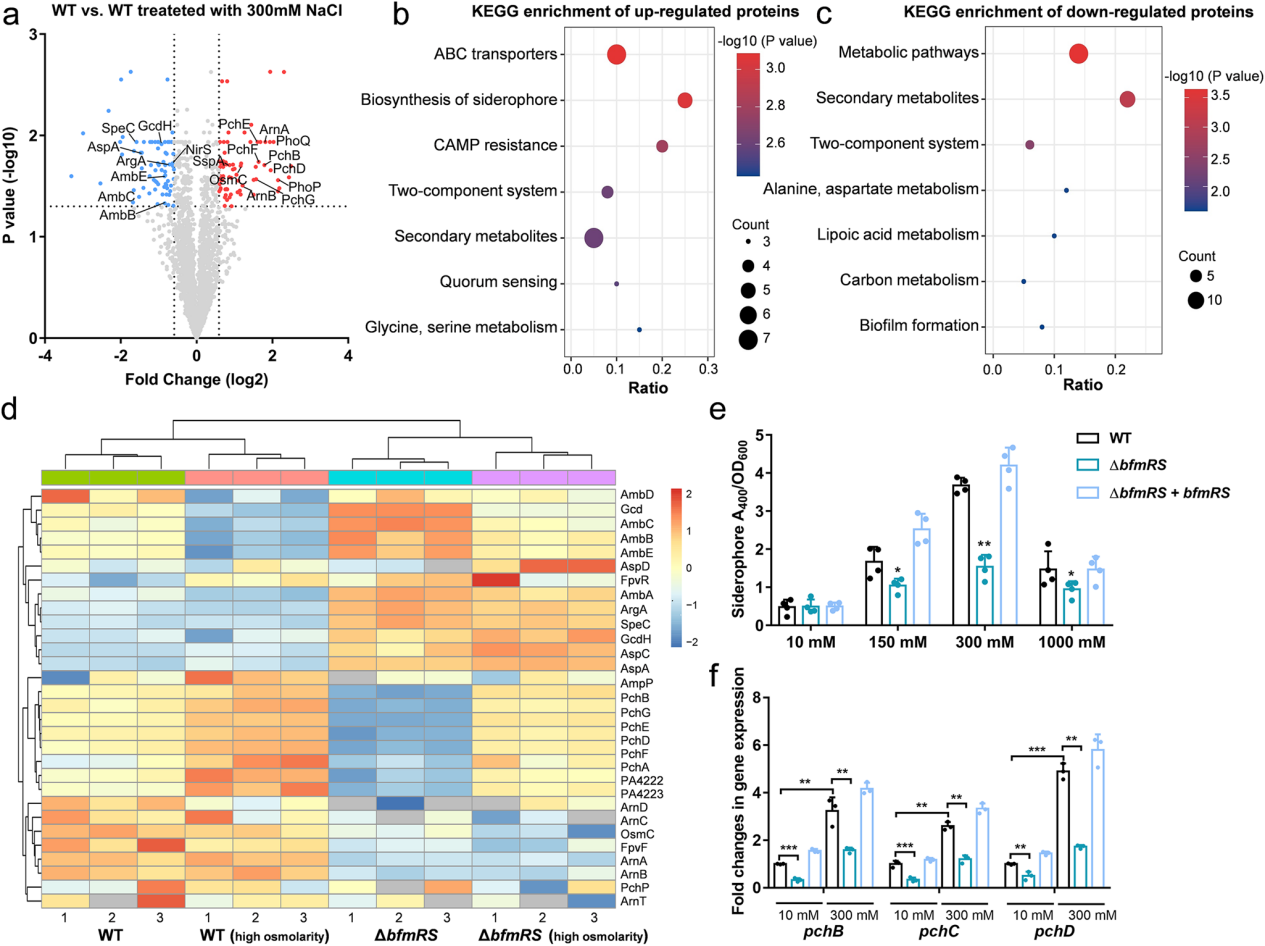
BfmRS contributes to siderophore metabolism in response to increasing osmolytes. **a** Volcano plot displaying the proteomic profiles of WT and WT treated with a high osmolarity medium. The significantly up- and down-regulated proteins are categorized by KEGG enrichment functional category as **b**, **c**, respectively. **d** Heatmap depicting the expression levels of proteins involved in siderophore metabolism, ion transport, antibiotic resistance, and amino acid metabolism among WT, WT with high osmolality, Δ*bfmRS*, and Δ*bfmRS* with osmolality. **e** Siderophore production of WT and mutants in various osmolyte media. **f** The expression changes of *pch* cluster genes in WT and mutant strains with or without high osmolality were measured. Error bars indicate the means ± SD of three independent experiments. *P < 0.05; **P < 0.01; ***P < 0.001.

Previously, upregulation of iron uptake genes in response to osmotic stress has been observed in *P. putida* and *Sinorhizobium meliloti*^21,32^. To further investigate the link between osmotic stress response and the dynamics of siderophore synthesis in *P. aeruginosa*, we conducted a proteomic analysis by comparing the DEPs of WT *P. aeruginosa* under high osmotic condition (grown in the presence of 300 mM NaCl) and normal growth condition. We observed a downregulation of proteins associated with amino acid metabolism and secondary metabolites in the WT strain exposed to high osmolality^33^ (Fig. 2a–c, Supplementary Data 2). In contrast, critical transporters and enzymes linked to siderophore biosynthesis, such as MlaE and PchB, displayed significant upregulation under high osmotic condition. Remarkably, the extent of their upregulation paralleled that of the osmotically inducible protein OsmC and polymyxin resistance proteins. Similar phenotypes were also observed in viable *E. coli*, the osmotically inducible gene *osmY* was specially activated by exogenous polymyxin B which could destroy the integrity of inner and outer membranes^34^. Thus, hyperosmotic stress response is considered a safeguard of bacteria against the polymyxin B to avoid the leakage of solutes and protons.

We further compared the protein expression profiles of the Δ*bfmRS* strain with the WT strain under high osmotic stress conditions. Our results revealed that BfmRS exerts positive regulation on many genes involved in siderophore synthesis (Fig. 2d). Conversely, proteins related to amino acid metabolism and the negative regulator of siderophore synthesis FpvR^35^, were negatively regulated by BfmRS (Fig. 2d, Supplementary Fig. 3). These observations suggest that the BfmRS system employs a unique regulatory mechanism for controlling siderophore metabolism in response to high osmotic pressure. In line with this, the Δ*bfmRS* strain showed a significant impairment in siderophore synthesis with increasing external osmolality. Specifically, at 150 mM and 300 mM NaCl conditions, we observed a 1.62- to 2.49-fold reduction in siderophore production in mutant strain compared to the parental or complementation strains (Fig. 2e). In contrast, the WT strain exhibited increased siderophore production with higher salt concentrations in the growth medium (Fig. 2e). All strains experienced a substantial decrease in siderophore production under 1 M NaCl condition, likely due to growth inhibition induced by excessive osmotic stress (Fig. 2e). Thus, the decreased siderophore production in the mutant strains may be attributed to a malfunctioning osmosensing mechanism. Additionally, we analyzed the mRNA levels of *pchB*, *pchC*, and *pchD* in response to increasing external osmolality. The results showed that the transcript levels of these genes were ~2–4.5-fold higher in the WT strain when exposed to high osmolality compared to low osmolality (Fig. 2f). Conversely, the upregulation of *pch* genes induced by high osmolality was notably impaired in the Δ*bfmRS* strain (Fig. 2f). In conclusion, these findings underscore the significant role of the BfmRS system in modulating siderophore production in *P. aeruginosa* in response to variations of external osmotic pressures.

### BfmR is a direct regulator of PVDI and iron metabolism genes

**Fig. 3:**
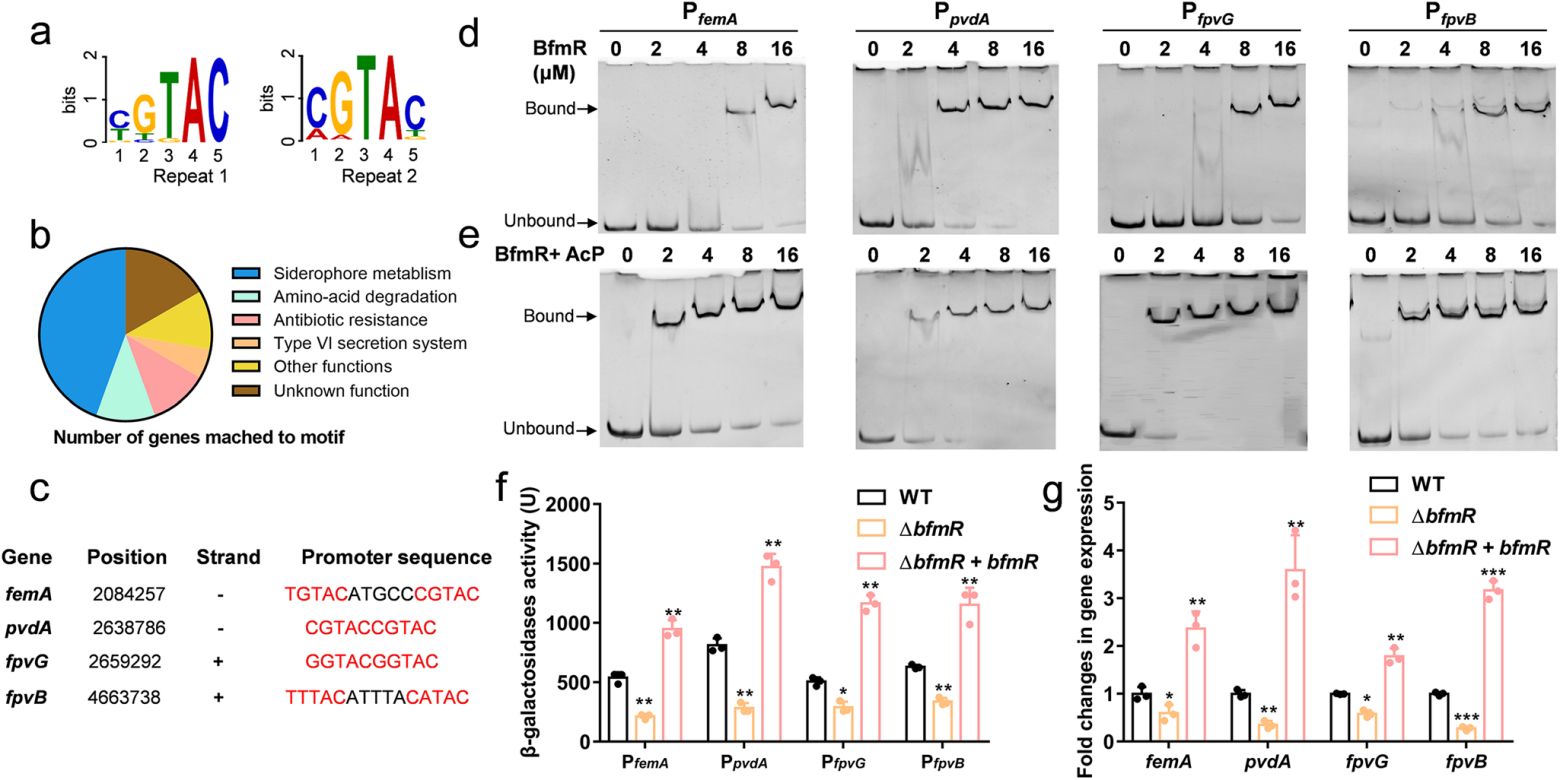
BfmR directly activates the expression of siderophore-associated genes. **a** The potential BfmR recognition motif. The sequence contains a direct repeat of two. 5-bp motifs separated by a 0 or 5 bp spacer, as CGTAC (N_0,5_) CGTAC. **b** Functional cluster analysis of genes predicted to be regulated by BfmR in *P. aeruginosa* PAO1. These loci were obtained by whole-genome screening based on BfmR recognition motif. **c** BfmR-binding sites in the promoter regions of selected siderophore genes. **d** EMSAs of BfmR with four gene promoters. The final DNA concentration was 1 μM, protein concentrations varied from 0 to 16 μM. BfmR and DNA fragments were preincubated at 4 °C for 30 min and electrophoresed on 8% polyacrylamide gel, then stained by EB dye and visualized under imager. **e** AcP treatment enhanced the DNA-binding ability of BfmR. The purified BfmR was treated with 10 μM acetyl phosphate (ACP) at 4 °C for 30 min and then applied for EMSAs. **f** Construction of β-galactosidase reporter system to determine the transcriptional activities of four targeted genes in *P. aeruginosa*. **g** Expression changes of four genes between WT, Δ*bfmR*, and genetically complemented strain (Δ*bfmR* + *bfmR*) (*n* = 3, *P < 0.05; **P < 0.01; ***P < 0.001. ns, no significance).

Approximately 199 putative motifs of regulators in *P. aeruginosa* were reported in a recent study, including BfmR, which contains a direct repeat of two 5 bp motifs (Fig. 3a)^36^. Based on this, we undertook a comprehensive whole-genome scanning against *P. aeruginosa* PAO1 genome, and there remained 20 potential binding loci of BfmR after screening (Supplementary Table 1). Further analysis showed that ~40% of these targeted genes played crucial roles in siderophore metabolism (Fig. 3b). Among these genes were *femA*, *pvdA*, *pvdD*, *fpvG*, and *fpvB* (Fig. 3c), which are responsible for PVDI synthesis, transport, and iron reduction^9^. The direct interactions between BfmR and the promoter regions of selected siderophore genes were also confirmed by performing electrophoretic mobility shift assays (EMSAs) (Fig. 3d), and no binding was observed between BfmR and DNA fragment lacking the BfmR-binding motif (Supplementary Fig. 4). Further experiments revealed that the presence of Acetyl phosphate (AcP) significantly enhanced the binding of BfmR to its target genes (Fig. 3e), suggesting that AcP could increase the activities of BfmR. We also measured the activities of these promoters fused with *lacZ* in WT and Δ*bfmR* strains. The loss of *bfmR* resulted in a significant decrease in promoter activities in vivo compared to the WT strain (Fig. 3f), with reductions ranging from 1.42- to 2.37-fold. Meanwhile, complementation of BfmR in the Δ*bfmR* strain significantly increased the promoter activities of these genes (Fig. 3f). A similar trend was also observed for the transcriptional levels of these genes in *P. aeruginosa* (Fig. 3g), providing further evidence that BfmR could directly activate the expressions of those genes in vivo.

**Fig. 4:**
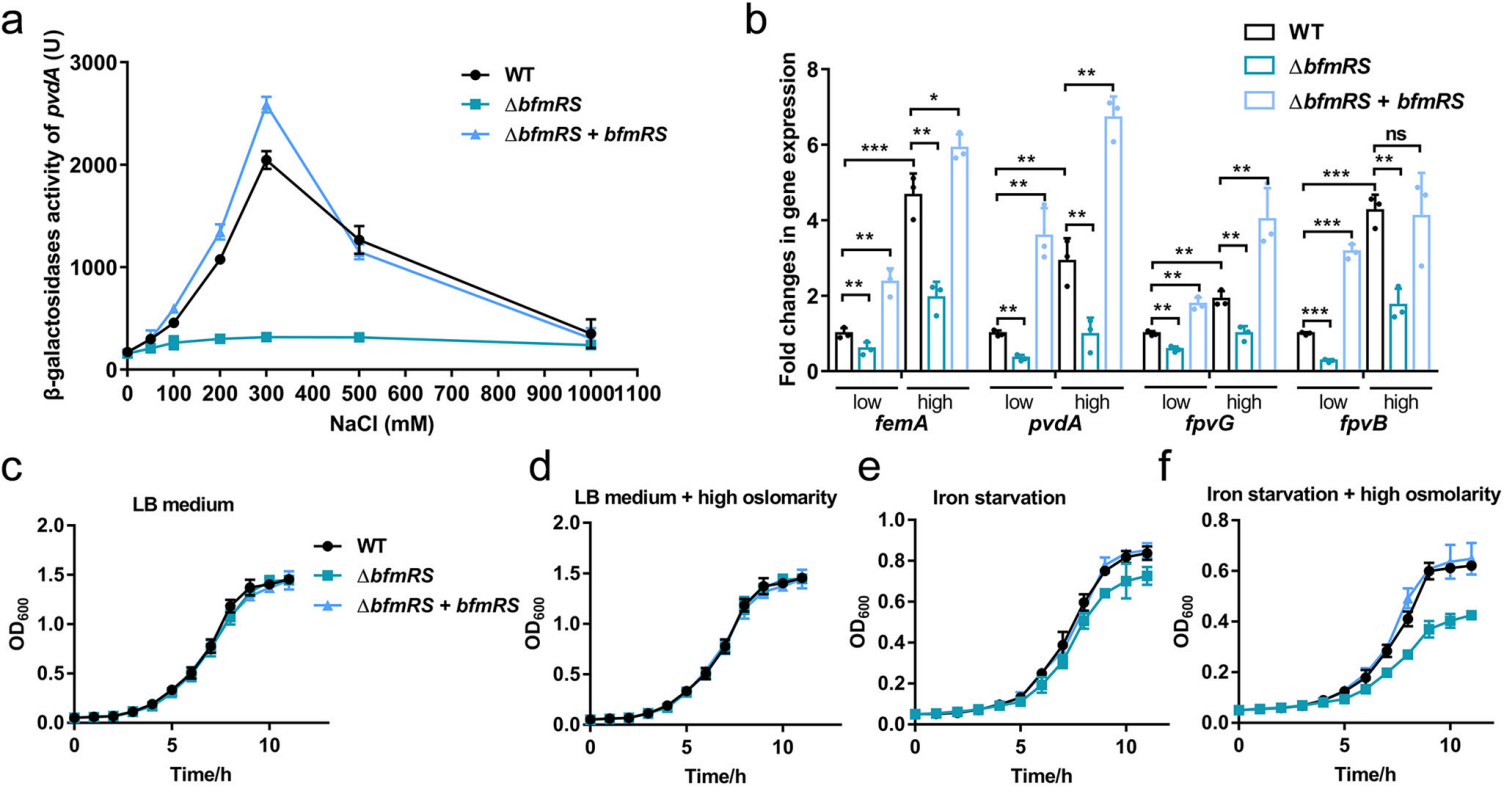
Effect of the BfmRS-mediated siderophore regulation on the growth of *P. aeruginosa* in response to osmotic stress. **a** BfmRS can sense osmolality and activate the expression of *pvdA*. β-galactosidase assays of the *pvdA*-*lacZ* transcriptional fusion were performed under diverse osmotic stresses. **b** Expression changes of *femA*, *pvdA*, *fpvG*, and *fpvB* in WT, Δ*bfmRS*, and complemented strain exposed to high osmolality compared to low osmolality. Growths of WT, Δ*bfmRS*, and Δ*bfmRS* + *bfmRS* were measured in LB medium (**c**), LB medium with high osmolality (**d**), iron deficiency medium with low osmolality (**e**), and iron deficiency medium with high osmolality (**f**). *P < 0.05; **P < 0.01; ***P < 0.001. ns, no significance.

We further conducted a β-galactosidase assay using a *pvdA*-*lacZ* transcriptional fusion to assess the impact of osmolality on gene expression in WT, Δ*bfmRS*, and Δ*bfmRS* + *bfmRS* strains following exposure to varying salt concentrations in the growth medium. In the WT strain, the promoter activities of *pvdA* displayed an increasing trend as ion concentration reached 300 mM (Fig. 4a), whereas minimal changes were observed in the Δ*bfmRS* strain (Fig. 4a). Similarly, the expression levels of *femA*, *pvdA*, *fpvG*, and *fpvB* were significantly upregulated in the WT strain under high osmolality, in contrast to low osmolality, while the absence of *bfmR* attenuated their activation (Fig. 4b).

Given that siderophores confer a competitive growth advantage to *P. aeruginosa*^7^, we then investigated whether BfmRS contributes to bacterial growth under conditions of iron deficiency and/or high osmotic pressure. In the presence of iron, no growth defect was observed under normal osmolarity condition or high osmolarity condition (Fig. 4c, d). However, the *bfmRS* deletion mutant exhibited slightly reduced growth compared to the WT strain in iron-limited medium (Fig. 4e). Importantly, the growth restriction caused by the deletion of *bfmRS* was more pronounced under iron deficiency condition combining high osmotic pressure (Fig. 4f). This suggests that BfmRS-mediated siderophore production activated by high osmotic pressure plays a significant role in *P. aeruginosa* growth under iron deficiency condition.

### Characterization of the DNA-binding domain of BfmR

The phosphorylation mechanism of BfmS has been well-studied based on missense mutations and other spontaneous variants from various clinical isolates of *P. aeruginosa*^26^. However, the DNA recognition process of BfmR is yet to be experimentally validated. Comparing BfmR with known TCSs RR, the high sequence similarity between BfmR and MtrA, PhoP, and RegX3 (MtrAB, PhoPB, and RegX3/SenX3 from *Mycobacterium tuberculosis*, sharing 34.8%, 33.04%, and 36.28% sequence identity, respectively), as well as DrrB and DrrD (OmpR/PhoB subfamily in *Thermotoga maritima*, with 31.25% and 34.78% sequence identity, respectively), and BaeR (BaeRS in *E. coli*, with 34.78% sequence identity) was noted (Supplementary Fig. 5a). Subsequently, we generated the full-length structure of BfmR, encompassing the N-terminal receiver domain and the C-terminal DNA-binding domain (DBD), using AlphaFold^37^ (Supplementary Fig. 5b). Similar to other regulatory domains within the RR superfamily, the N-terminal region of BfmR adopts an α/β sandwich topology, comprising a central five-stranded parallel sheet surrounded by α helices on each side. The C-terminal region of BfmR represented a winged-helix domain responsible for DNA recognition (Supplementary Fig. 5b,c).

**Fig. 5:**
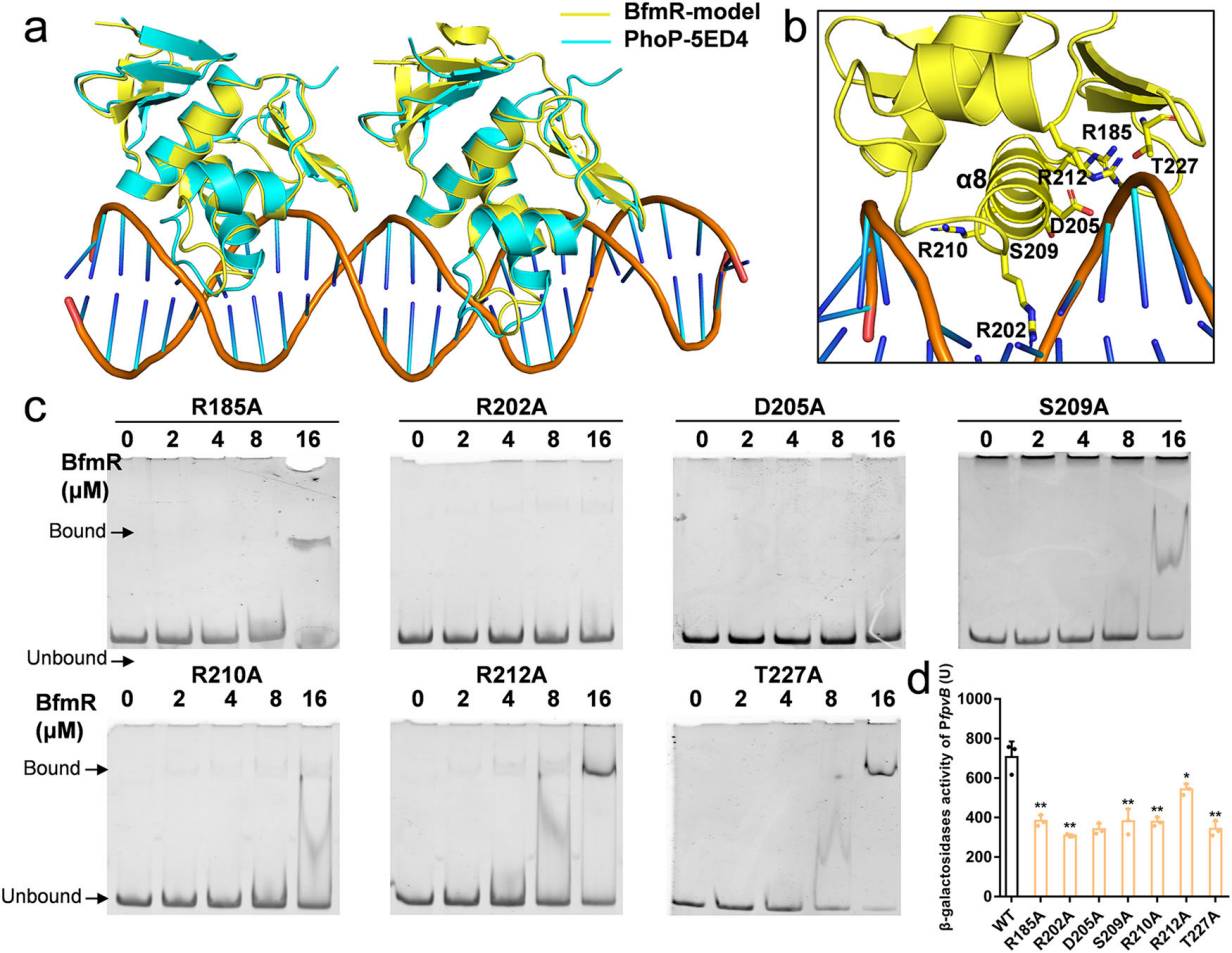
Structural model of BfmR-DNA complex. **a** Ribbon diagram of the BfmR-DNA complex showing a dimer of BfmR DBD binding to the promoter DNA. The model was generated by the superposition the BfmR DBD with the PhoP-DNA structure. **b** Detailed interaction between DBD and DNA, the residues involved in DNA recognition are shown in sticks. **c** EMSAs of different mutant BfmR proteins with *fpvB* promoter. The final DNA concentration was 1 μM, protein concentrations varied from 0 to 16 μM. BfmR and DNA fragments were preincubated at 4 °C for 30 min and electrophoresed on 8% polyacrylamide gel, then stained by EB dye and visualized under imager. **d** β-galactosidase reporter system to determine the transcription regulation ability of BfmR mutants. *P < 0.05; **P < 0.01; ***P < 0.001.

Furthermore, a BfmR-DNA model was constructed based on the structure of *M. tuberculosis* PhoP in complex with DNA (PDB: 5ED4)^38^. In this model, two copies of BfmR DBDs adopt a tandem head-to-tail arrangement, binding to the double-helix DNA (Fig. 5a). Each α8 helix on the DBD inserts into the DNA groove, facilitating the formation of a compact complex (Fig. 5a). Residues R185, D205, S209, R212, and T225 directly interacted with the DNA phosphate backbone, while R202 and R210 extended into the major groove of the DNA, interacting with the bases (Fig. 5b). Sequence alignment of BfmR with its homologs revealed the high conservation of R185, R212, and T227 among these proteins, with D205 and S209 being moderately conserved (Supplementary Fig. 5a). On the other hand, the residues corresponding to R202 and R210 exhibited significant variations among these proteins, suggesting their potential role in determining the specificity of the targeted DNA sequence (Supplementary Fig. 5a). Consistent with the BfmR-DNA structure, alanine substitutions of R185, R202, D205, S209, R210, and T227 nearly abolished the ability of BfmR to recognize the P_*fpvB*_ in the EMSAs (Fig. 5c). Although R212A substitution retained partial DNA-binding capacity, its binding affinity was significantly reduced compared to the WT (Fig. 5c). Furthermore, the circular dichroism spectra for WT and mutant proteins were detected, and no significant change of the proportions of α-helixes and β-sheets was found between WT and mutants, suggesting that the changes of DNA-binding abilities were due to the amino acid replacement (Supplementary Fig. 5d). This was aligned in the reduced transcriptional activation activities of P_*fpvB*_ in β-galactosidase reporter assays (Fig. 5d). These results not only lend further support to the molecular model of BfmR but also underscore the critical residues in DNA recognition.

### The role of BfmRS in siderophore coordination for *P. aeruginosa* virulence

**Fig. 6:**
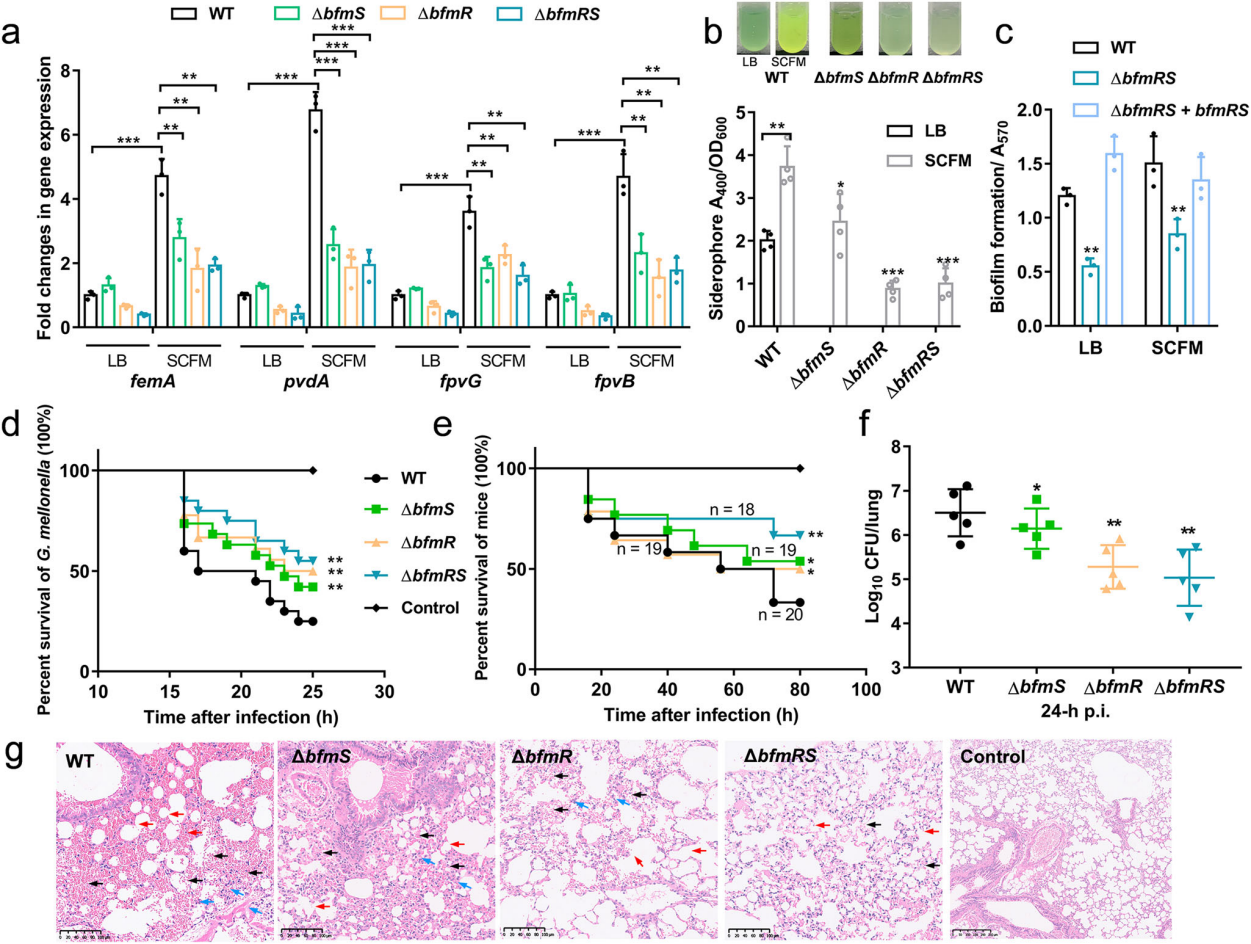
*bfmS* or *bfmS* deletion impaired bacterial virulence and the ability of *P. aeruginosa* to adapt to the host. **a** The expression changes of *femA*, *pvdA*, *fpvG*, and *fpvB* in WT and mutant strains grown in SCFM medium. **b** Siderophore and (**c**) biofilm production of WT and mutants in SCFM medium. **d** Measurement virulence of the WT and mutant strains in a *G. mellonella* infection model. Each *G. mellonella* was injected with 10 µL of *P. aeruginosa* dilution (1 × 10^4^ CFU/mL), and the PBS-injected larvae were the negative control. The larvae were monitored for 25 h after the infection (Mantel–Cox test for statistics, *P < 0.05). **e** Survival of mice infected with WT or mutants, **P < 0.01 (log-rank test; all mutants compared with WT). In the murine acute pneumonia model, each mouse was infected intranasally with 1 × 10^7^ CFU of WT or mutants. 24 h post infection, mice were sacrificed, and bacterial loads in the lungs were determined (**f**). **g** For histopathology analysis, H&E-stained sections of lungs infected with WT or mutants were viewed at a magnification of ×100. Blue arrows indicate neutrophil infiltration, black arrows indicate extravasated blood in lung, and red arrows indicate damage to alveolar structure. Scale bars, 50 µm.

Pathogens, such as *Pseudomonas species*, rely on siderophores to acquire iron from host tissues, making siderophores crucial for virulence^7,10,12,13^. Previous studies have demonstrated that the high levels of Na^+^ and Cl^-^ in respiratory tract fluid of CF patients can stimulate alginate synthesis during infection^39^. To investigate the role of BfmRS-mediated siderophore production for *P. aeruginosa* virulence, we utilized a synthetic cystic fibrosis medium (SCFM) to examine the impact of BfmRS on siderophore metabolism under conditions resembling CF sputum^40^. The results, depicted in Fig. 6a, highlighted a robust upregulation of BfmR-targeted gene expression (3.82- to 6.74-fold) when compared to growth in standard conditions. Notably, this upregulation significantly diminished in both Δ*bfmS* and Δ*bfmR* mutants. Furthermore, the upsurge in gene expression in the WT strain correlated with significantly enhanced siderophore production when cultivated in the SCFM medium, as depicted in Fig. 6b. The increased production of siderophores was notably absent in the Δ*bfmS*, Δ*bfmR*, and Δ*bfmRS*, reinforcing the role of BfmRS in coordinating siderophore synthesis under CF-mimicking conditions (Fig. 6b). Additionally, we reaffirmed the importance of the BfmRS system in biofilm formation. As shown in Fig. 6c, the lack of *bfmRS* expression resulted in impaired biofilm development compared to the WT strain in SCFM medium, underscoring its multifaceted role in *P. aeruginosa* pathogenicity.

Additionally, survival studies conducted in *Galleria mellonella* larvae and a murine pneumonia infection model further emphasized the impact of BfmRS on pathogenesis. The loss of *bfmS* or *bfmR* in *P. aeruginosa* exhibited reduced mortality rates in *G. mellonella* larvae at 16 h, with death rates of 37.9% and 25.0%, respectively (Fig. 6d). Meanwhile, the WT group exhibited near 50% mortality (Fig. 6d). Similarly, infections with the WT strain led to a 70% mortality rate in infected mice within 80 h. However, Δ*bfmR* and Δ*bfmRS* displayed a delay in mortality, resulting in 50% and 75% survival rates, respectively (Fig. 6e). Additionally, at 24 h post infection, the mutants exhibited significantly lower bacterial loads in the lungs compared to the WT strain (Fig. 6f). Histological examinations of lung sections (HE staining) revealed intense neutrophil infiltration, extravasated blood in the lungs, and alveolar structure damage in the WT strain, whereas milder inflammation was observed in infections caused by the mutants (Fig. 6g, Supplementary Fig. 6). These findings strongly support the notion that the BfmRS system significantly contributes to *P. aeruginosa*’ s ability to better adapt to the host environment, facilitating the establishment of long-term infections.

### Ubiquitous distribution and conserved siderophore regulation mechanism of BfmRS homologs in *Pseudomonas* species

**Fig. 7:**
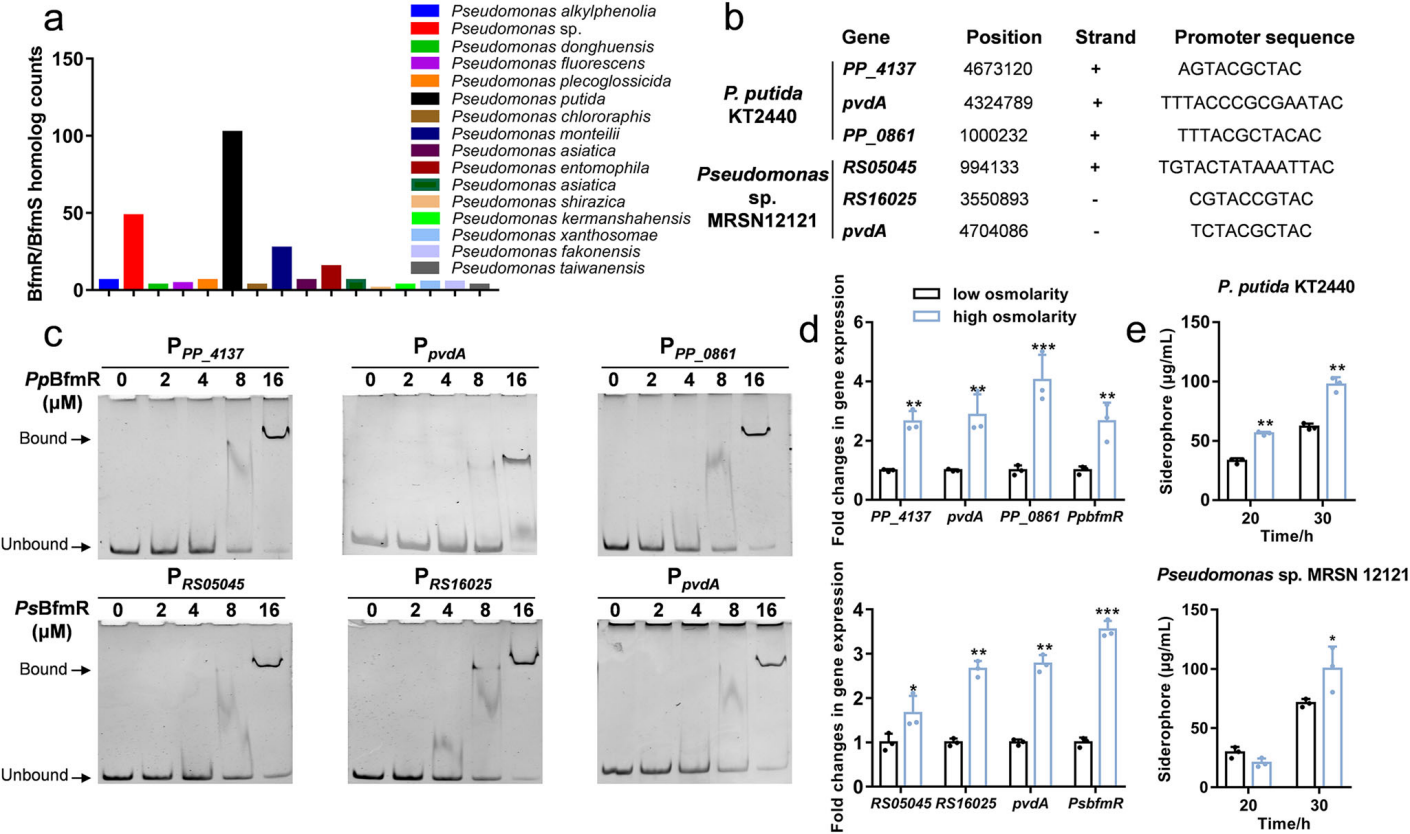
BfmRS system is highly conserved and widely distributed across *Pseudomonas* species. **a** Distribution of BfmRS homologs in 16 representative *Pseudomonas* species. The total number of BfmRS homologs in different genomes of *Pseudomonas* species is shown as *y* axis. This statistic shows the number of *Pseudomonas* strains containing BfmRS homologs, and no duplicate BfmRS system found in the same bacterial strain. **b** Potential binding loci of BfmR homologs in *P. putida* KT2440 and *Pseudomonas* sp. MRSN12121 (abbreviated as *Pp*BfmR and *Ps*BfmR, respectively), and the direct interactions were further confirmed by EMSAs (**c**). The final DNA concentration was 1 μM, protein concentrations varied from 0 to 16 μM. **d** Relative mRNA levels of targeted genes in *P. putida* KT2440 and *Pseudomonas* sp. MRSN12121 in different osmolyte media. The *oprL* gene was used as a normalizer. **e** Siderophore production of *P. putida* KT2440 and *Pseudomonas* sp. MRSN12121 in different osmolyte media. *P < 0.05; **P < 0.01; ***P < 0.001.

To ascertain the functional ubiquity of the conserved BfmRS TCS within the *Pseudomonas* genus and their association with siderophore metabolism, we conducted a search for BfmRS homologs in the *Pseudomonas* Genome DB^41^. Using the DIAMOND BLASTX search tool with specific parameters (E-value cut-off 1e-12, 70% query coverage, and 50% identity cut-off), we identified a mount of BfmRS homologs in 16 *Pseudomonas* species, including *Pseudomonas fluorescens*, *Pseudomonas plecoglossicida*, *P. putida*, *Pseudomonas monteilii*, and *Pseudomonas* sp., typically prevalent as pathogens in animals or plants (Fig. 7a). Notably, *P. putida* and *Pseudomonas* sp. exhibited a higher abundance of BfmRS systems compared to other *Pseudomonas* species. Moreover, the BfmR homologs displayed high conservation with BfmR from *P. aeruginosa*, particularly in conserved residues responsible for DNA recognition (Supplementary Fig. 5 and 7), implying the existence of common DNA-binding motifs.

To determine the direct regulatory role of BfmR homologs on siderophore genes, a DNA motif based on *P. aeruginosa* BfmR was utilized to scan potential binding loci within the genomes of *P. putida* KT2440 and *Pseudomonas* sp. MRSN12121. Figure 7b illustrated that similar binding sites were identified in both genomes, encompassing genes associated with siderophore production, including the *pvdA* gene. EMSAs confirmed the direct binding between BfmR homologs and their respective target genes (Fig. 7c). Furthermore, transcriptional analysis revealed increased expression levels of *bfmR* homologs, *pvdA*, and other siderophore-associated genes in both of *P. putida* KT2440 and *Pseudomonas* sp. MRSN12121 under high osmolality (Fig. 7d). These findings were in line with a substantial increase in siderophore production observed under high osmolality condition (Fig. 7e). Collectively, these results suggest that the BfmRS TCS represents a pivotal mechanism governing siderophore regulation and is widely conserved across diverse *Pseudomonas* species.

## Discussion

**Fig. 8:**
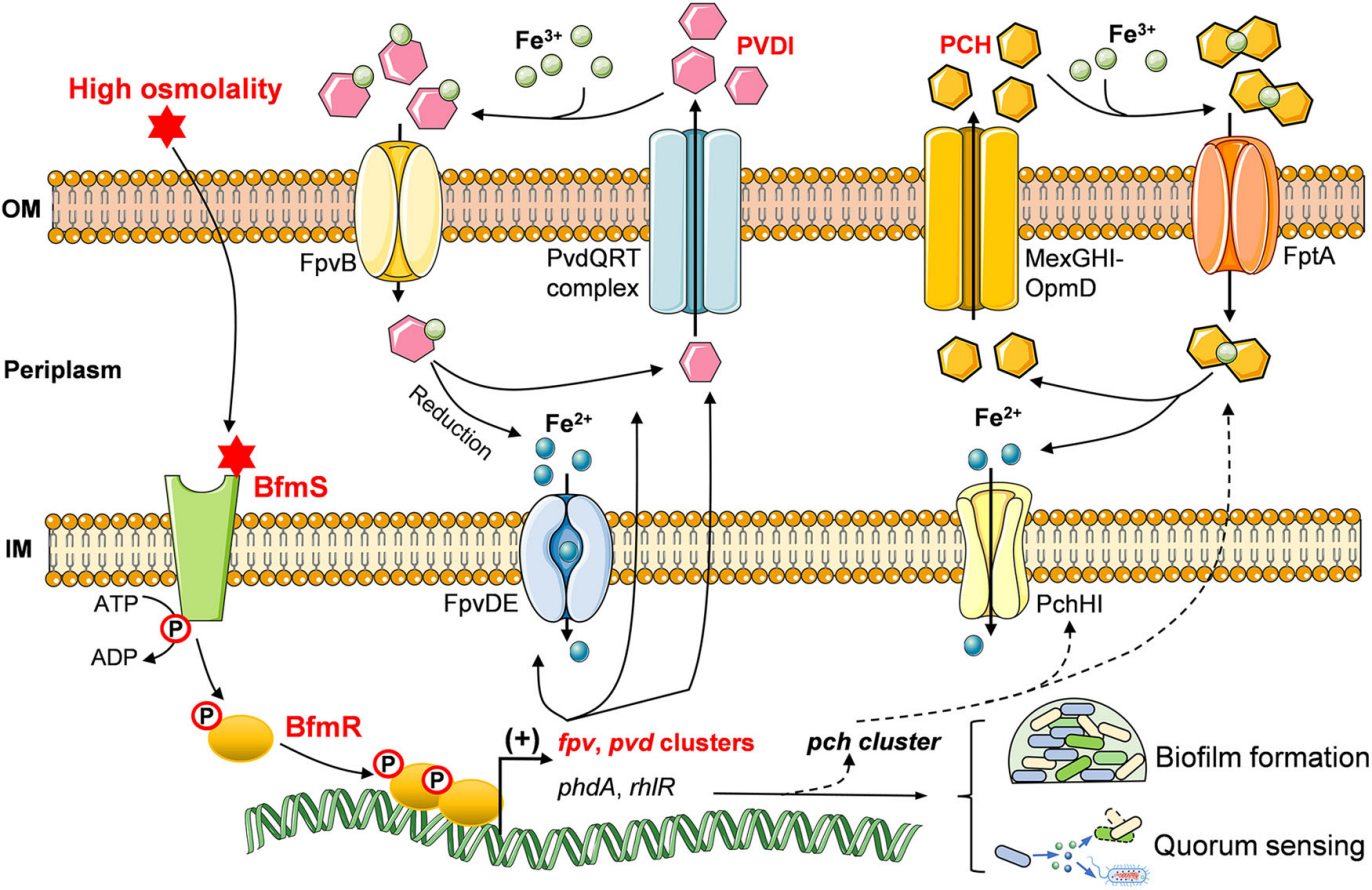
Schematic representation of BfmR/BfmS-based siderophore regulation in *P. aeruginosa*. The HK BfmS undergoes autocatalytic phosphorylation on a conserved histidine residue in response to increasing osmolytes, then the phosphoryl group is transferred to RR BfmR (BfmR~P). BfmR~P specifically binds to the promoter regions of *fpv*, *pvd*, and *femARI* clusters, to activate their transcription, leading to a high level of PVDI. On the other hand, BfmR could also modulate the *rhl* QS system and biofilm formation by regulating the expression of *phdA* and *rhlR*, respectively. The increased production of PCH may be attributed to the BfmRS-mediated QS regulation. Parts of the figure were drawn by using pictures from Servier Medical Art, provided by Servier, licensed under a Creative Commons Attribution 3.0 unported license (https://creativecommons.org/licenses/by/3.0/).

TCSs enable bacteria to adapt their cellular processes and virulence in response to various environmental stimuli^16,42^. However, our understanding of bacterial TCSs remains limited, even in well-studied organisms like *P. aeruginosa*, a commonly isolated microbe in patients with bloodstream and lung infections in hospitals. In this study, we have discovered a new signal-transduction pathway, BfmS/BfmR/ (*pvd*, *fpv*), which plays a crucial role in regulating siderophore metabolism in *P. aeruginosa*. We have demonstrated that BfmRS belongs to the OmpR/EnvZ family and is activated under high osmolarity. BfmR directly binds to the promoter regions of *pvd*, *fpv*, and *femARI* clusters, enhancing their expression. Deletion of either *bfmR* or *bfmRS* resulted in a significant reduction in siderophore production and impaired bacterial survival in a mouse lung infection model. Furthermore, we have found that BfmRS is highly conserved in other *Pseudomonas* species and similarly associated with siderophore metabolism. Based on these findings, we proposed a model illustrating the signal-transduction mechanism of BfmRS TCS, as depicted in Fig. 8.

In previous studies, the multifaceted role of BfmRS in various pathogens has been documented. BfmR has been shown to be crucial for pellicle and biofilm formation in *Acinetobacter baumannii* and *P. aeruginosa*^24,27^. Additionally, it plays a pivotal role in the bacterium’s survival at the mammalian host temperature in multiple animal models, contributing significantly to chronic infections^25,26,29^. It has been reported that BfmR has a potential extensive downstream regulatory network, and BfmS influences the tolerance to certain antibiotics, outer membrane vesicle (OMV) production, and cytotoxicity toward host cells^43^. Studies have also begun to shed light on the signals sensed by BfmS, such as light or pressure^29^. Despite these advances, a comprehensive understanding of its complete functional repertoire and its intricate regulatory mechanisms in complex biological processes remains an area necessitating further exploration. Therefore, our study aimed to conduct a more in-depth dissection of this TCS, highlighting novel findings of BfmRS in essential metabolic pathways in *P. aeruginosa*. The comprehensive examination of BfmRS revealed that BfmR directly interacts with the promoter regions of critical siderophore genes to constitute a BfmS/BfmR/ (*pvd*, *fpv*) signaling cascade in response to osmolarity changes. Contrary to Δ*bfmS*, deletion of *bfmR* caused a significant decrease of siderophore production in normal condition, and a previous study also proved that P_*bfmR*_ exhibited higher activities in Δ*bfmS* or missense mutation strains found in the CF-adapted isolates^26^. Given that HK proteins can serve both as a net kinase and net phosphatase whose activities depend on the status, our results further indicate that BfmS determines the regulatory activity of BfmR in response to extracellular clues, representing an unusual strategy of adaptive evolution in bacterial pathogens. This finding paves the way for comprehensive investigations into similar TCSs and their impact on microbial pathogenicity and survival mechanisms, laying the groundwork for potential therapeutic interventions in bacterial TCSs.

Adaptation to high osmolality environments is a fundamental aspect observed across diverse domains of life, from Bacteria and Archaea to Eukarya, representing an evolutionary conserved trait^30,44^. The extensively studied OmpR/EnvZ system has been notably recognized for its pivotal role in responding to changes in external osmolality^30^. For instance, in *E. coli*, the OmpR/EnvZ TCS is known to perceive increasing osmolarity and activate the promoters of *ompC* and *ompF*, genes encoding outer membrane proteins that are instrumental in controlling the passive diffusion of small hydrophilic molecules across the cell membrane^45^. Recent studies have further expanded the scope of the OmpR/EnvZ TCS, revealing its broader influence beyond the regulation of OmpC/OmpF porins^30^. It is implicated in diverse pathogens like *Shigella flexneri*, *Salmonella typhimurium*, *Yersinia pestis*, *A. baumannii*, and *Shewanella oneidensis*, where it also controls phase variation, motility, and virulence^46^. This implies that osmoregulatory responses enable bacteria to dynamically adapt to challenging environments. Notably, osmolarity also constitutes a significant host environmental factor affecting bacterial responses^39,45^. For example, the respiratory tract fluid of CF patients contains elevated levels of Na^+^ and Cl^-^ ions compared to healthy individuals^39^, our findings underscore the critical role of the BfmRS system in *P. aeruginosa* infections, particularly in latent and chronic lung infections. Furthermore, considering the higher osmolality in the rhizosphere soil water influenced by solute exclusion of plant roots, osmotic pressure becomes an essential stimulus for successful colonization of rhizobacteria in the rhizosphere^47^. As the BfmRS system is widespread in certain *Pseudomonas* species found in rhizobacterial, more in-depth study can be performed to fully elucidate the precise functional mechanism of this system. Future investigations should be focused on understanding how bacteria utilize this type of TCS to sense high environmental osmolarity and regulate their own metabolism, virulence, and drug resistance mechanisms accordingly.

Iron is indispensable for the survival of *P. aeruginosa* as well as other bacteria and can be obtained through various sources under aerobic conditions, including its own siderophores PCH and PVDI, xenosiderophores, or heme^7,13,14^. Commonly, the production of siderophore is initially triggered by iron shortage and regulated by the master regulator Fur, however, emerging evidences also demonstrated that other environmental clues stimulate the siderophore synthesis processes. In *P. putida*, osmotic stress-induced changes in membrane composition decrease permeability^48^. This included the upregulation of cardiolipin biosynthetic genes and numerous transporters, and downregulation of several other transporter-related proteins. Interestingly, an upregulation of iron uptake mechanisms (siderophores) was also observed during osmotic stress, highlighting the need for iron as a cofactor for various enzymes activated during stress. A similar phenotype was also observed in *E. coli*, in which the OmpR/EnvZ TCS responds to increasing medium osmolarity and thereby enhances the intracellular pool of accessible iron^22^. Our research builds on these foundations to further elucidate the complex iron metabolism regulation in *P. aeruginosa* and offers a molecular explanation for the link between osmotic stress and iron metabolism regulation. Our proteomics analysis also revealed an upregulation of siderophore metabolism in response to osmolality in the WT strain but not *bfmRS* mutant. This finding suggests a sensitive molecular switch for iron regulation in *P. aeruginosa*.

While all fluorescent *Pseudomonas* species are known to produce major siderophores called PVDI, questions regarding the ecological and systematic regulation of siderophore metabolism in this bacterial genus have only recently gained attention. Although several TCSs have been implicated in iron uptake processes in *P. aeruginosa*, their impact on siderophore metabolism is limited^16^. We demonstrated that BfmR directly activates the promoters of *fpv*, *pvd*, and *femARI* gene clusters in response to high osmolality. Interestingly, loss of *bfmRS* also caused significant downregulation of *pch* genes compared to WT, but no direct binding was observed between BfmR and promoter region of *pch* cluster (Supplementary Fig. 8). This may be triggered by other cascade regulations such as QS system due to the direct regulation of BfmR on *rhlR*, an essential regulator of QS system^24^. Thus, further study is needed to uncover the whole regulation network of BfmRS in *P. aeruginosa*. Similarly, the homologous OmpR/EnvZ system in *E. coli* was found to maintain iron homeostasis by regulating the FeoB-mediated iron transport pathway. Deletion of *ompR* or porin genes significantly impaired bacterial growth under iron-depleted conditions, similar to the growth defect observed in the *bfmRS* mutant. However, unlike OmpR, which exerts its effects on ferrous iron regulon in part with Fur, we identified the BfmRS system as a signal-transduction cascade involved in siderophore metabolism in *Pseudomonas* species. Moreover, it is worth noting that numerous ABC transporters, TCSs, and metabolic enzymes exhibited significant expression changes between the *bfmRS* mutant and WT strains under high osmolality, suggesting that the BfmRS TCS may also play a role in other pathways.

Overall, this study provides valuable insights into the multifaceted functions of the BfmRS system in response to osmolality stress, highlighting the role of BfmR as an activator of siderophore metabolism for stress adaption in *P. aeruginosa*. Our findings underscore the significance of BfmRS in bacterial growth under iron depletion conditions and adaption to the lung environment during infections. We emphasize the potential applicability of BfmRS-mediated siderophore regulation as a stress adaptation strategy in other bacteria, given the high conservation of the BfmRS TCS across *Pseudomonas* species. Considering the wide range of applications of siderophores and their derivatives in ecology, agriculture, bioremediation, biosensors, and medicine—such as enhancing soil fertility, bio-controlling fungal pathogens, detoxifying samples contaminated with heavy metals, and enabling selective drug delivery^9,10^—the *bfmRS* operon represents a promising genetic element for improving siderophore applications. We anticipate that further studies focusing on elucidating the molecular mechanisms and comprehensive regulatory networks of BfmRS will significantly contribute to the understanding of how this system functions in *Pseudomonas* species.

## Methods

### Bacterial strains, plasmids, and growth conditions

Bacteria strains and plasmids used in this work are listed in Supplementary Table 2. All mutants were constructed by gene allelic substitution as described in the Methods. *P. aeruginosa* PAO1 and its derivatives were grown on LB medium (10 mM NaCl for low osmolality, 150 mM for normal condition, and 300 mM for high osmolality), Succinate minimal medium (40 mM succinate, 9.3 mM NH_4_Cl, 2.2 mM KH_2_PO_4_, 25 mM KNO_3_, 25 mM NaNO_3_, 30 mM MOPS (pH 7.2)) at 37 °C with shaking (220 r/min).

### Construction of *P. aeruginosa* Δ*bfmS*, Δ*bfmR*, and Δ*bfmRS* mutant strains

For gene replacement, a gene allelic substitution based on *sacB* was used to construct Δ*bfmS*, Δ*bfmR*, and Δ*bfmRS* mutants^49^. The upstream (800 bp) and downstream (800 bp) fragments of *bfmR*, *bfmS,* and *bfmRS* genes from the *P. aeruginosa* PAO1 were subcloned into the plasmid pEX18Gm, respectively. Then the recombinant plasmids were transformed into *P. aeruginosa* PAO1 via *E. coli* S17-1. The colonies were firstly screened by gentamicin sensitivity (unable to survive on LB agar plates containing 50 µg/mL gentamicin) and loss of sucrose sensitivity (survive on LB agar plates containing 10% w/v sucrose), then identified by PCR. All the primers used in this work are listed in Supplementary Table 3.

### Bioinformatics analysis

To explore the distribution of BfmRS homologs across diverse bacterial taxa, the Uniprot Blast was used to search the BfmRS homologs, and the top 500 hits were selected (for both HK and RR in the same operon, coverage >70%, identify >40%). These were filtered to remove species without a complete genome, to ensure that genome incompleteness did not influence system detection. As for the known EnvZ proteins, the amino acid sequences were obtained from Uniprot database, and the sequence alignments were prepared using Clustal Omega (https://www.ebi.ac.uk/Tools/msa/clustalo) online server. The phylogenetic trees of BfmRS and BfmS were generated from PhyML 3.1/3.0 aLRT (http://www.phylogeny.fr/) via the maximum likelihood method and refined graphically using ITOL v6 (https://itol.embl.de/) with default parameters.

### Mass spectrometry sample preparation and measurement

Total proteins of WT and Δ*bfmRS* were collected, and purified by using a Bacterial Protein Extraction Kit (Jiangsu Enzymes Biotechnology), and the concentration of samples was measured by Bicinchoninic Acid Assay. Extracted proteins (50 μg) from each sample were firstly treated by 1 mL of pre-cooled acetone at −20 °C for 14 h, then redissolved and reduced by 8 M urea buffer containing 10 mM dithiothreitol, and further alkylated by 25 mM dithiothreitol in darkness. 10 *k*Da ultrafiltration centrifugal device was used to exchange the urea buffer for buffer containing 50 mM ammonium bicarbonate. The protein samples were digested by 1 μg sequence grade trypsin (Promega) at 37 °C for 16 h and then pooled with 10% trifluoroacetic acid buffer (0.4% v/v final concentration). The peptides of each sample were combined and applied for reversed-phase HPLC (Vanquish, Thermo Fisher Scientific) under basic pH, with a mobile phase consisting of buffer 1 (2% acetonitrile, pH = 10) and buffer 2 (98% acetonitrile, pH = 10). A total 43 min LC gradient (comprising of buffer 1 and buffer 2, flow rate was 1 mL/min) was used as: 0–5 min, 2–2% buffer 2; 5–7 min, 2–5% buffer 2; 7–27 min, 5–18% buffer 2; 27–37 min, 18–32% buffer 2; 37–38 min, 32–90% buffer 2; 38–40 min, 90% buffer 2; 40–41 min, 90–2% buffer 2; 41–43 min, 2% buffer 2. The peptide mixture was separated into 39 fractions and combined into 13 fractions. All peptides were desalted and dried in the speed vacuum (Christ).

### LC-MS/MS analysis

The obtained peptides were resuspended by buffer 3 (0.1% formic acid), and iRT peptides (Bignosys) were supplied to correct the retention time (RT) of peptides. All peptide samples were applied on a homemade capillary column (75 μm i.d. × 25 cm, ReproSil-Pur C18-AQ, 1.9 μm; Dr. Maisch) and tested via LC-MS/MS using EASY-nLC 1200 system coupled to an Orbitrap Exploris 480 mass spectrometer (Thermo Fisher Scientific). The mobile phase consisting of buffer 3 (0.1% formic acid) and buffer 4 (80% acetonitrile, 0.1% formic acid) was used to elute the samples at 300 nL/min with a gradient: 0–2 min, 3%–8% buffer 4; 2–54 min, 8–28% buffer 4; 54–68 min, 28%–40% buffer 4, 68–70 min, 40%–100% buffer 4, 70–78 min, 100% buffer 4. All the peptide samples were acquired using the data-independent acquisition (DIA) method, the full scan was performed between 350 and 1500 m/z with 60,000 resolutions, with the settings (300% AGC target and 50 ms maximum injection time). Then 45 DIA windows were scanned (350–1500 m/z) with a resolution of 15,000 where precursor ions were fragmented with NCE set at 30% and analyzed with AGC target of 500% and auto maximum injection time.

### Mass spectrometry data analyses

The obtained raw data for samples were processed via Spectronaut against the in-house library with the default settings. The optimal extraction window was dynamically determined by the Spectronaut based on gradient stability and iRT calibration. The retention time was predicted based on dynamic iRT. The mass tolerance for both MS1 and MS2 was set to dynamic. Identifications for peptides and proteins were matched to protein sequences from *P. aeruginosa* PAO1 obtained from Uniprot Proteome database and further processed through the software Perseus. As for statistical analysis, the student’s *t* test was used to test the DEPs of WT and Δ*bfmRS*. Threshold of DEPs as *p* value < 0.05 and fold change ≥1.5 or ≤0.67.

### Siderophore assay

*P. aeruginosa* WT and Δ*bfmS*, Δ*bfmR*, and Δ*bfmRS* mutants were grown in LB overnight at 37 °C, then 1 mL culture of each sample was centrifuged at 12,000 rpm for 2 min and resuspended in the iron-deficient succinate medium and grown for 48 h^49^. For siderophore measurement, the supernatants were filtered through 0.45 μm PVDF syringe filter (Millipore), and the absorbance at 400 nm was measured. Meanwhile, the OD_600_ of cultures was also measured, and the final siderophore production was calculated as A_400_/OD_600_. As for *P. putida* KT2440 and *Pseudomonas* sp. MRSN12121, the bacteria were grown on a similar medium, and the supernatants were filtered through 0.45 μm PVDF syringe filter (Millipore) and used for Chrome Azurol S (CAS) assay^50^. 1 mL supernatant of bacterial cultures was mixed with equivalent CAS buffer and cultured at 16 °C for 1 h, a color change from blue to orange was read at the absorbance 630 nm using UV Spectrophotometer, with blank culture medium as control. The standard curve was established with the standard PVDI samples (Sigma-Aldrich).

### Protein expression and purification

The *E. coli* BL21 (DE3) strains carrying pET22b-*bfmR* were cultured in LB medium at 37 °C until reaching an OD_600_ of 0.8. Then, 0.4 mM IPTG was added to the cultures for further shaking at 16 °C overnight. The cells were harvested and resuspended in a buffer consisting of 25 mM Tris-HCl pH 8.0, 150 mM NaCl, 2 mM Phenylmethanesulfonylfluoride, and 5% glycerol. Ultrasound was used to lyse the cells, and the resulting lysate was centrifuged at 14,000 rpm for 30 min. The supernatant was applied to Ni-agarose resin and eluted with a buffer containing 300 mM imidazole. The obtained proteins were subsequently purified using an AKTA purifier (GE Healthcare) and concentrated to a final concentration of 3 mg/mL. All the mutant proteins were purified using the same method and detected by circular dichroism spectra (Chirascan plus). Briefly, the protein samples were diluted to 0.1 mg/mL in buffer containing 25 mM Tris-HCl pH 8.0, 150 mM NaCl, and placed at 25 °C for 6 h. Then 200 μL sample was taken in a colorimetric dish (1 mm) for testing. The given results were the average of at least 3 parallel detections, followed by background and air deductions.

### Electrophoretic mobility shift assays

The promoters of the targeted genes were cloned from the genome of *P. aeruginosa* PAO1. For binding assays, BfmR and the DNA fragment were mixed in various ratios in a total volume of 10 µL. The mixture contained a buffer comprising 25 mM Tris-HCl pH 8.0, 150 mM NaCl, 1 mM MgCl_2_, and 2 mM KCl. The samples were then incubated at 4 °C for 0.5 h and analyzed by 8% polyacrylamide gel electrophoresis using 0.5× TBE (Tris/boric acid/EDTA) buffer at a voltage of 130 V for 2 h. The bands were visualized by staining with ethidium bromide (EB) and observed using a BioRad imager. As for AcP treatment, the purified BfmR was treated by 10 μM acetyl phosphate (ACP) at 4 °C for 0.5 h and then applied for EMSAs.

### Search for potential BfmR-binding loci

The DNA motif NNTAC (*N*_0,5_) NNTAC was used to blast the potential BfmR-binding loci in *P. aeruginosa* PAO1 genome with the help of DNA Motif Searches Tool in *Pseudomonas* Genome DB (https://www.pseudomonas.com/search/sequences). Based on preference from previous report, the short DNA motif prefers to be NNTAC (CG/GG) TAC^36^. In initial blast, >400 hits were found, most of them located in the gene coding regions. The results were further screened to remove the repeats or hits located in gene-coding regions or far away from promoter regions (>300-bp upstream of protein-coding genes). Due to the few gene function annotations in *P. putida* KT2440 and *Pseudomonas* sp. MRSN12121 genomes, the siderophore-associated clusters, were selected to screen potential BfmR-binding loci with similar DNA motif. Then, the results were further screened to remove the repeats or hits located in gene-coding regions or far away from promoter regions (more than 300-bp upstream of protein-coding genes).

### RNA extraction and real-time quantitative PCR (RT-qPCR)

Overnight bacterial cultures of WT and Δ*bfmR* were subcultured in fresh LB medium to OD_600_ 0.8, and the cells were collected and treated by Trizol (Invitrogen, USA) for 10 min. The cDNA was synthesized by using PrimeScript RT reagent Kit (TaKaRa, Beijing). 2× ChamQ SYBR qPCR Master Mix (Vazyme, Nanjing) was carried out to perform qPCR assays. The reaction mixture (20 µL) consisted of 2× ChamQ SYBR qPCR Master Mix (Without ROX) 10 µL, forward primer (10 µM) 0.4 µL, reverse primer (10 µM) 0.4 µL, cDNA 1 µg, and ddH_2_O. The qPCR procedure was performed as: step 1 (95 °C 30 s), step 2 (95 °C 10 s, 60 °C 30 s, repeat for 40 times), and step 3 (95 °C 15 s, 60 °C 1 min, 95 °C 15 s). The *oprL* gene was used as a normalizer.

### β-galactosidase assay

The promoter regions of *bfmR*, *femA*, *pvdA*, *fpvG*, and *fpvB*, ranging from 200 to 400 base pairs, were cloned upstream of a promoterless *lacZ* gene within the pRG970km vector, resulting in the construction of *lacZ* reporter plasmids. The pRG970km vector was then introduced into *P. aeruginosa* PAO1 through mediation by *E. coli* S17-1, and the transformed cells were selected on LB agar plates with 100 µg/mL kanamycin. The bacterial cultures were grown in LB medium at 37 °C until reaching an OD_600_ of 0.6. Subsequently, 1 mL culture was harvested and treated with 50 µL 0.1% sodium dodecyl sulfate buffer, and the galactosidase activities were measured by incubating 200 µL supernatant with 40 μL O-nitrophenyl-β-d-galactopyranoside (ONPG) buffer (4 mg/mL) for 10–30 min at 25 °C^49^. The β-Galactosidase could catalysis the colorless ONPG into yellow O-nitrophenol, so the absorptions at 420 nm and 550 nm were detected. The β-Galactosidase activity Miller Units (*U*) = 1000 × [(*A*_420_−1.75 × A_550_)]/(*T* × *V* × OD_600_). *T* = time of the reaction (min). *V* = volume of culture used in the assay (mL).

### Murine lung infection

Overnight bacterial culture was diluted 1:100 in fresh LB medium and grown at 37 °C until the OD_600_ reached 0.8. Then the cells were collected and washed twice with sterile phosphate-buffered saline (PBS). The bacterial cell concentration was adjusted to 5 × 10^8^ CFU/mL in sterile PBS. 20 µL bacterial solution was infused into the respiratory tract of C57BL/6 mice using nasal drip method for each mouse. At 40 h post infection, mice were euthanized via CO_2_ inhalation, and the lungs were applied for histological examinations. Additionally, equal weight lung tissues of different groups were homogenized in sterile PBS, diluted, and further counted on the LB agar plates.

### Ethics statement

Eight-week-old female C57BL/6 mice were purchased from Chengdu Dashuo Bioscience Company and housed in the experimental animal house of West China School of Public Health, Sichuan University. All animal experiments were reviewed and approved by the Animal Ethics and Safety Review Committee of Sichuan Normal University (Permit Number: 2023LS037) and carried out in compliance with institutional guidelines concerning animal use and care at Sichuan Normal University.

### Statistics and reproducibility

The data collected were analyzed using GraphPad Prism 7.0 software and presented as the mean ± standard error of the mean. Each experiment was conducted with a minimum of three independent biological replicates. When comparing two groups, such as mRNA levels, a two-tailed Student’s *t* test was employed. For the β-galactosidase, siderophore production, and biofilm formation assays, a one-way ANOVA with Bonferroni correction for multiple comparisons was utilized. In mouse infection assays, the Mantel-Cox test was performed for statistical analysis. Statistical significance was determined at a threshold of *P* < 0.05.

### Reporting summary

Further information on research design is available in the Nature Portfolio Reporting Summary linked to this article.

### Supplementary information

Supplementary information

Description of Additional Supplementary Files

Supplementary Data 1

Supplementary Data 2

Supplementary Data 3

Reporting Summary

## Data Availability

The MS raw files and proteome data that support the findings of this study are available in ProteomeXchange Consortium with the dataset identifier PXD046481^51^. The detailed DEPs of proteome data are presented as Supplementary Data 1 and 2. The source data underlying Figs. 1–7, Supplementary Figs. 2 and 5 are presented as Supplementary Data 3, whereas the uncropped gel/HE images are presented as Supplementary Fig. 9.
