# Supplementary material for: Molecular mechanism of siderophore regulation by the Pseudomonas aeruginosa BfmRS two-component system in response to osmotic stress
Source: Commun Biol. 2024 Mar 9;7:295. doi: 10.1038/s42003-024-05995-z (PMC10924945; doi:10.1038/s42003-024-05995-z)
Supplement: Supplementary file 1 — Supplementary information [file 42003_2024_5995_MOESM1_ESM.pdf]

Supplementary information

Molecular mechanism of siderophore regulation by the *Pseudomonas aeruginosa* BfmRS two component system in response to osmotic stress

Yingjie Song[1, #], Xiyu Wu[2, #], Ze Li[3], Qin qin Ma[1], Rui Bao[3]

[1]College of Life Science, Sichuan Normal University, Chengdu 610101, China;

[2]Advanced Mass Spectrometry Center, Research Core Facility, Frontiers Science Center for Disease-related Molecular Network, West China Hospital, Sichuan University, Chengdu 610213, China.

[3]Center of Infectious Diseases, Division of Infectious Diseases in State Key Laboratory of Biotherapy, West China Hospital, Sichuan University, Chengdu 610041, China;

[#]These authors contributed equally: Yingjie Song, Xiyu Wu.

Correspondence should be addressed to e-mail: Rui Bao: baorui@scu.edu.cn (R.B.).

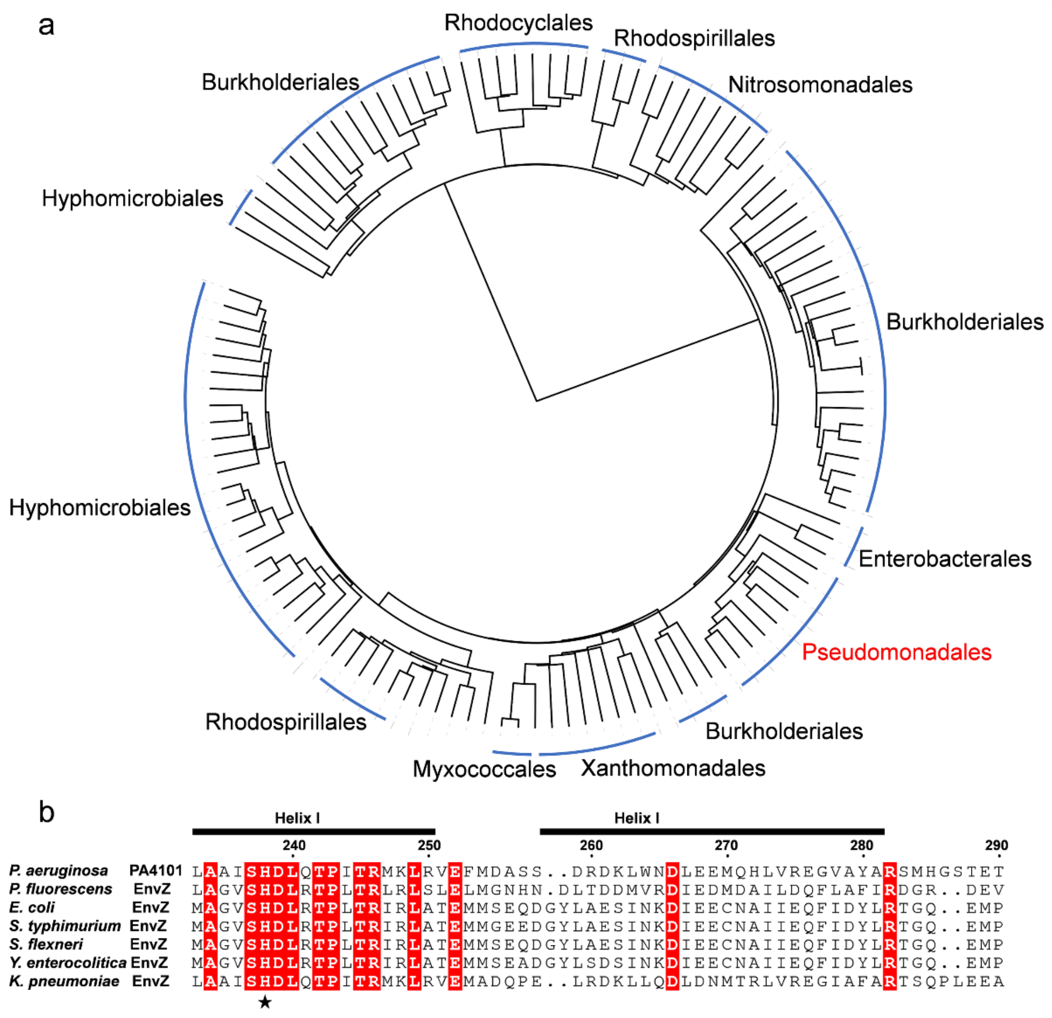

Supplementary Figure 1. BfmRS represents an OmpR/EnvZ type TCS across diverse bacteria. (a) Distribution of BfmRS homologs across diverse bacterial taxa. A bacterial phylogeny was retrieved from Uniprot blast for species containing at least one system in our homology search. These were filtered to remove species without a complete genome, to ensure that genome incompleteness did not influence system detection. The Pseudomonades phylum is coloured red. (b) Part of sequence alignment on BfmS and other known EnvZ proteins from *Pseudomonas fluorescens*, *Escherichia coli*, *Salmonella typhimurium*, *Shigella flexneri*, *Yersinia enterocolitica*, *Klebsiella pneumoniae*. The conserved histidine residue for autophosphorylation is labeled with solid asterisk.

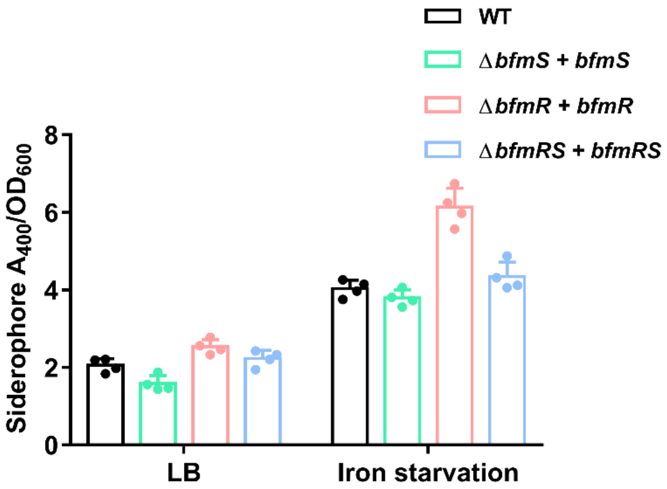

Supplementary Figure 2. Siderophore production of complementation strains. The complementation strains were constructed by transferring pRK415 containing each gene in these deletion mutants. The siderophore production was measured as described in methods.

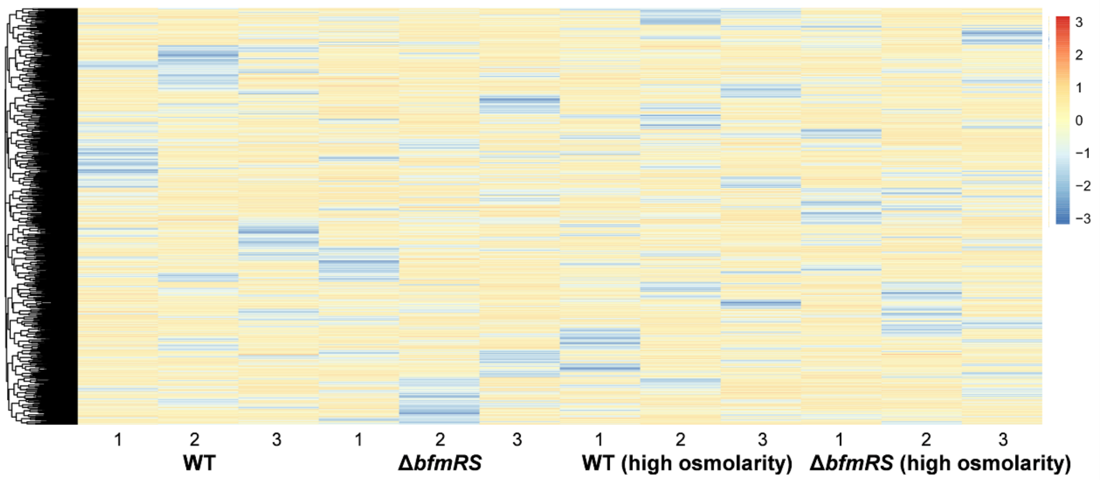

Supplementary Figure 3. Heatmap showing the protein changes of WT and ΔbfmRS with or without high osmolarity treatment. Hierarchical clustering of the z-scored extracted ion chromatogram was used to evaluate the reproducibility of the proteome quantification in WT and mutant.

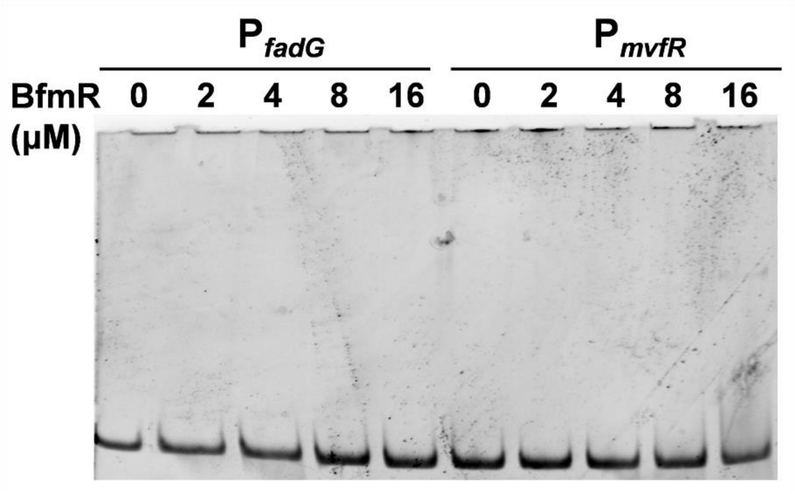

Supplementary Figure 4. BfmR specially recognized promoter regions of siderophore-associated genes. There was no interaction between BfmR and other promoters without BfmR motif. The final DNA concentration was 1 μM, protein concentrations varied from 0 to 16 μM.

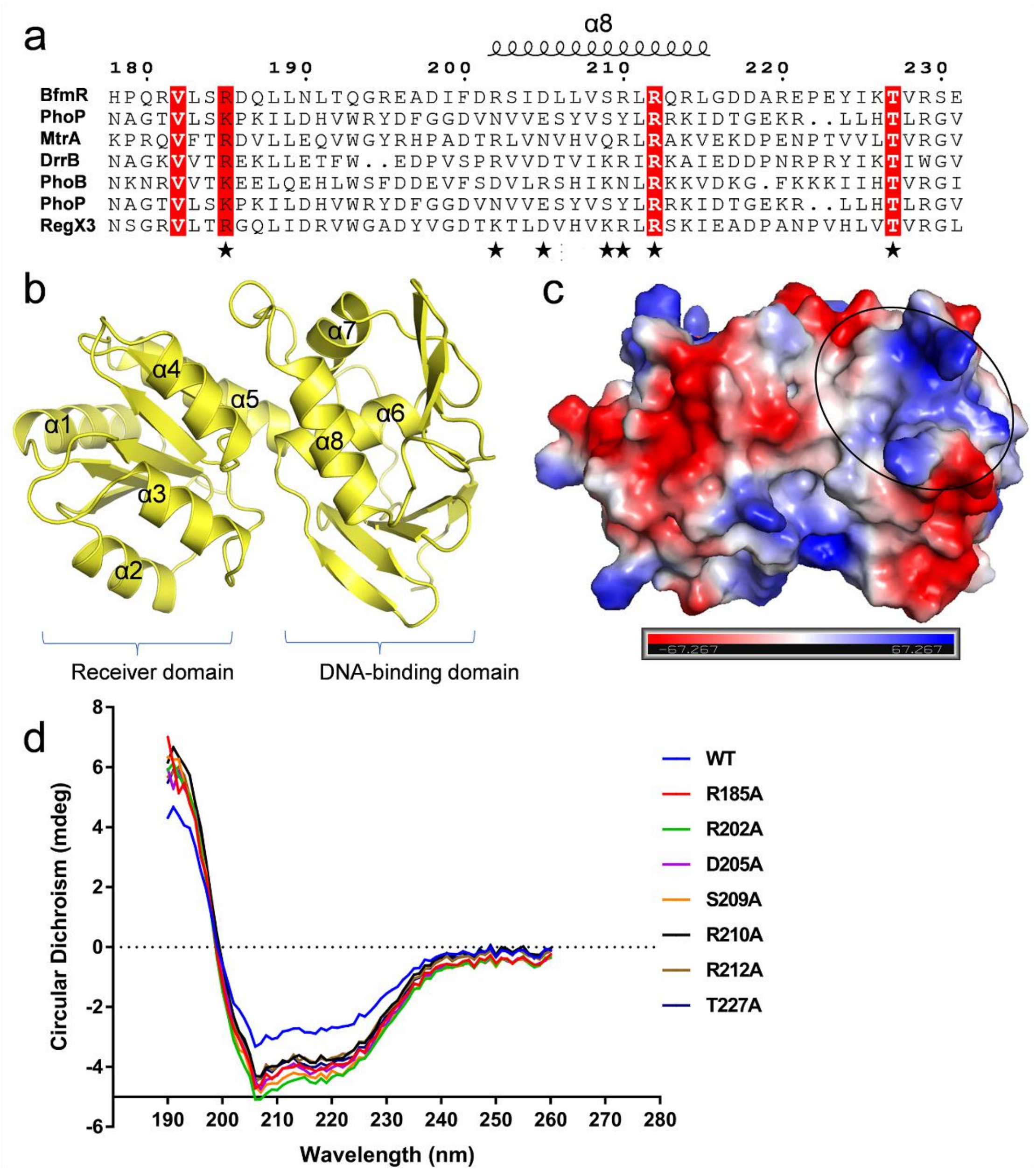

Supplementary Figure 5. Structural model of BfmR-DNA complex. (a) Part of sequence alignment on BfmR with homologous regulators. The potential residues involved in DNA interaction are labeled with solid asterisk. (b) Cartoon presentation of the BfmR monomer. The BfmR consist of a N-terminal receiver domain and a C-terminal DBD with positively charged surface (c). (d) The circular dichroism spectra for WT and mutant proteins were detected, and the proportions of α-helixes and β-sheets were calculated.

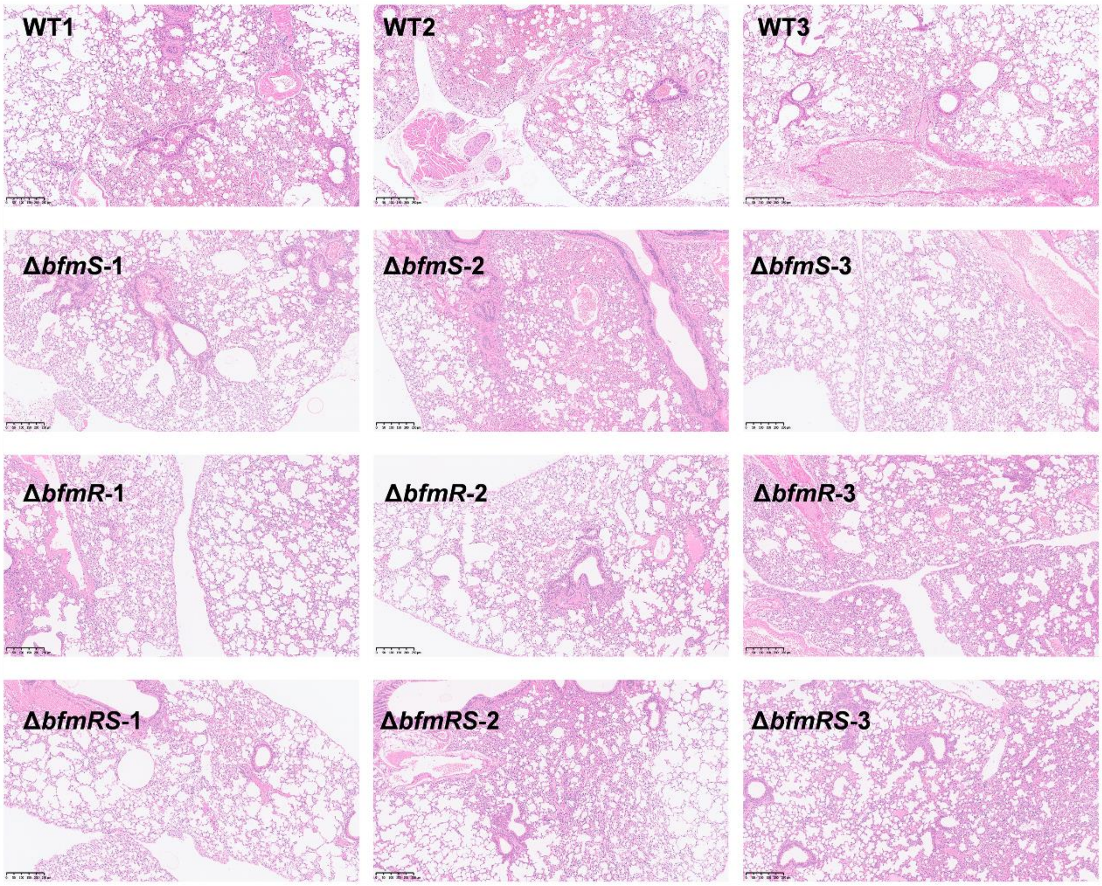

Supplementary Figure 6. Additional H&E-stained sections of mice lungs infected with WT and mutants. The lung tissues were viewed at a magnification of × 100.

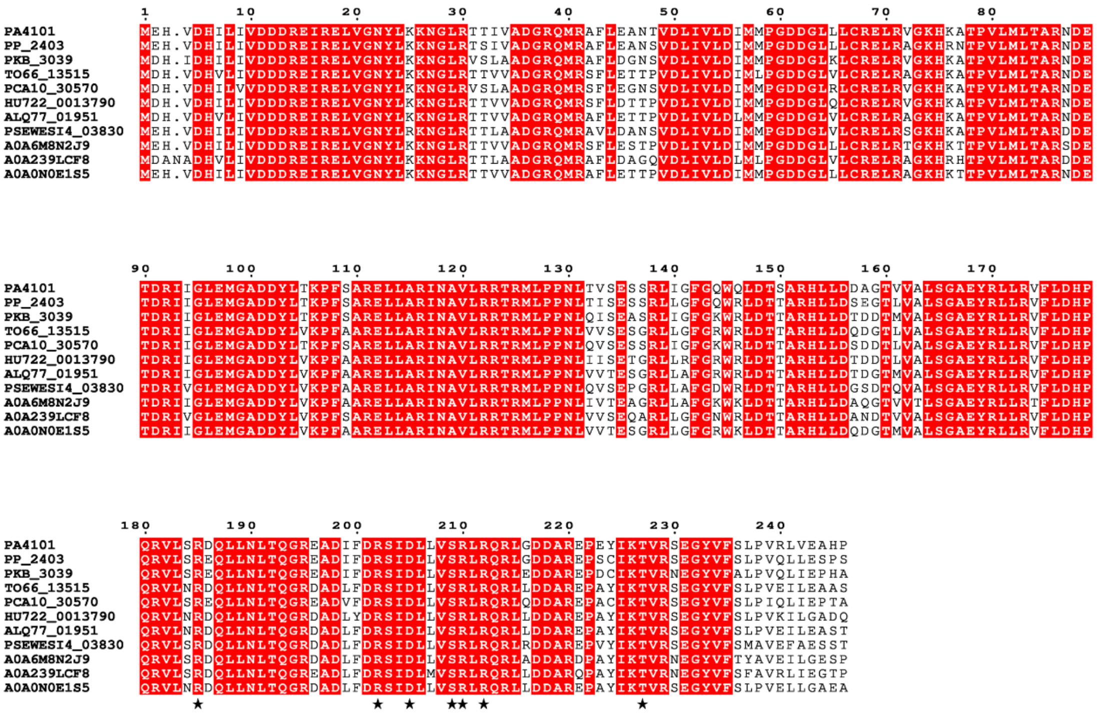

Supplementary Figure 7. The residues of BfmR involved in DNA interaction are highly conserved among homologs from *Pseudomonas* species. Sequence alignment of BfmR (PA4101) and homologs, including PP2403 from *P. putida*, PKB_3039 from *Pseudomonas knackmussii*, TO66_13515 from *Pseudomonas* sp. MRSN 12121, PCA10_30570 from *Pseudomonas resinovorans*, HU722_0013790 from *Pseudomonas tritici*, ALQ77_01951 from *Pseudomonas corrugate*, PSEWESI4_03830 from *Pseudomonas carbonaria*, A0A6M8N2J9 from *Pseudomonas graminis*, A0A239LCF8 from *Pseudomonas japonica*, and A0A0N0E1S5 from *Pseudomonas fuscovaginae*. The potential residues involved in DNA interaction are labeled with solid asterisk.

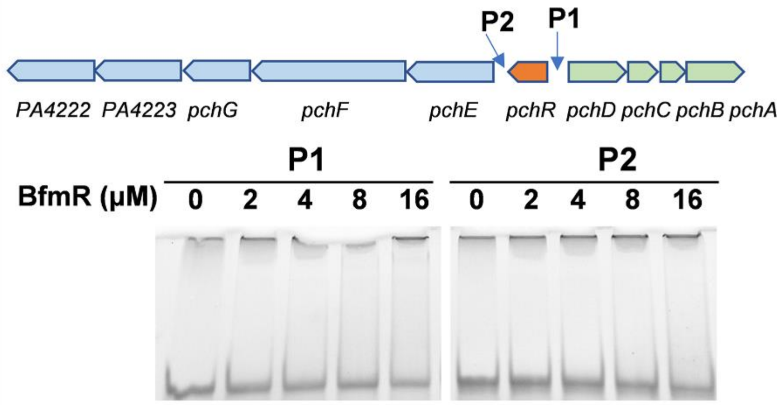

Supplementary Figure 8. BfmR couldn't bind to the promoter regions of *pch* cluster. The final DNA concentration was 1 μM, protein concentrations varied from 0 to 16 μM. The organization of *pch* cluster is shown in upper panel.

Supplementary figure 9. Uncropped and unedited gel/HE images.

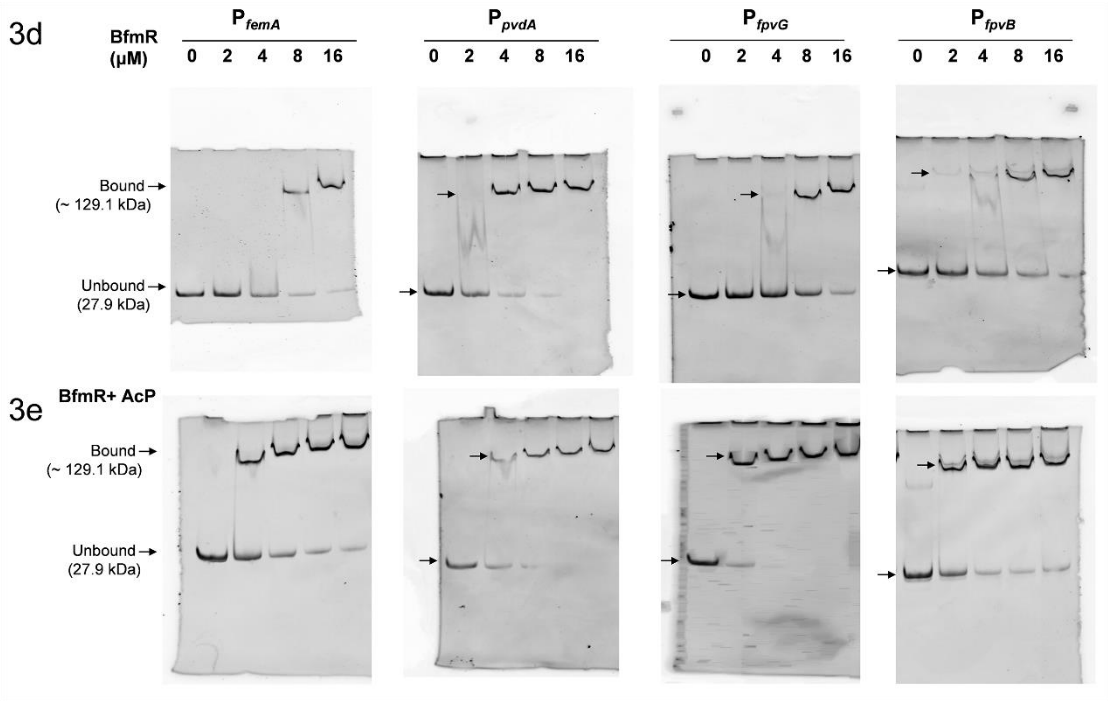

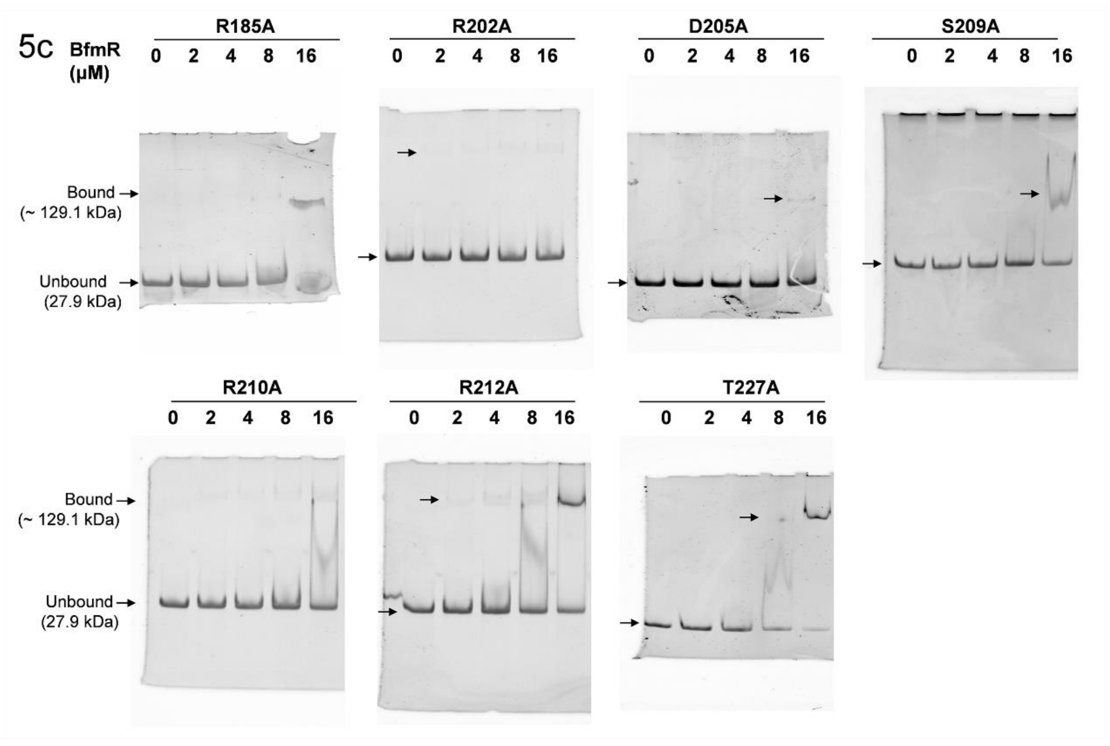

6g

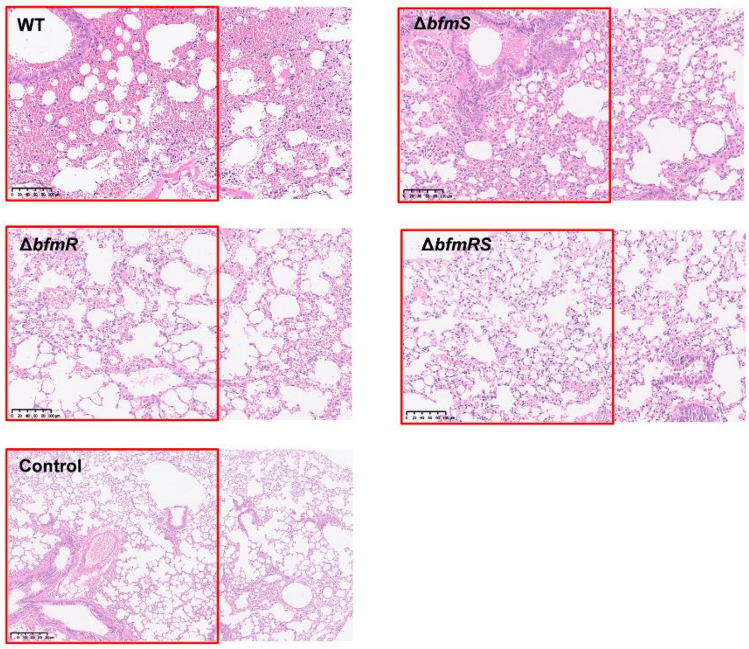
WT
ΔbfmS
ΔbfmR
ΔbfmRS
Control

7c

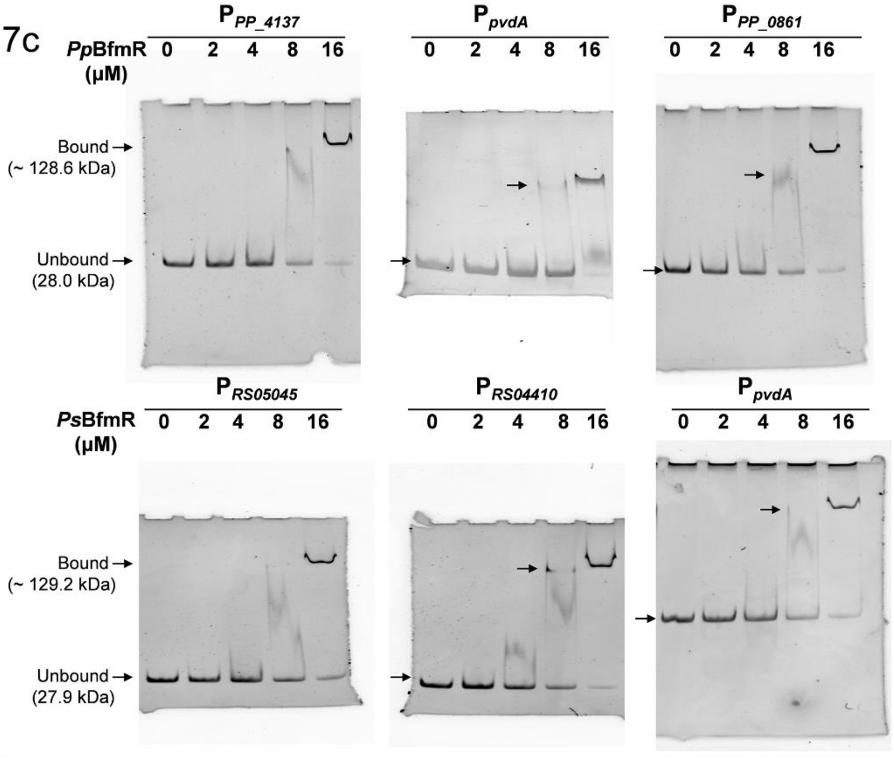
P_PP_4137
P_pvdA
P_PP_0861
PpBfmR (μM)
0 2 4 8 16
Bound
(~ 128.6 kDa)
Unbound
(28.0 kDa)
P_RS05045
P_RS04410
P_pvdA
PsBfmR (μM)
Bound
(~ 129.2 kDa)
Unbound
(27.9 kDa)

Supplementary Table 1. Potential BfmR-binding loci in *P. aeruginosa* PAO1 genome.

Gene	Function	Position start	Position end	Sequence	Strand
PA0435	unknown	501220	501229	CCTACCGTAC	-
PA0500 (BioB)	Biotin synthase	559477	559491	ACTACTGGCTTCTAC	+
PA0902	unknown	985642	985656	CCTACGGCGCTCTAC	+
PA2601	LysR family transcriptional regulator	2945227	2945234	GCTACCGTAC	-
PA1286	MFS transporter	1397226	1397240	TCTACGGGTATATAC	
PA1328	unknown	1441005	1441014	AGTACGGTAC	-
PA1660 (*hsiG2*)	virulence	1808896	1808905	TGTACTGTAC	+
PA1871 (*lasA*)	virulence	2033571	2033580	CGTACCGTAC	-
PA1910 (*femA*)	siderophore	2084257	2084271	TGTACATGCCCGTAC	-
PA2399 (*pvdD*)	siderophore	2659292	2659301	TGTACCGTAC	+
PA2424 (*pvdL*)	siderophore	2708386	2708395	TGTACCGTAC	-
PA2386 (*pvdA*)	siderophore	2638786	2638795	AGTACGGTAC	+
PA2399 (*pvdD*)	siderophore	2659292	2659301	CGTACCGTAC	+
PA2403 (*fpvG*)	siderophore	2687482	2687491	GGTACGGTAC	+
PA2423 (*pvdL*)	siderophore	2708386	2708395	GGTACGGTAC	+
PA3449	unknown	3854923	3854932	TATACGGTAC	+
PA4101 (*bfmR*)	biofilm formation	4584930	4584944	GATACATGGCCGTAC	+
		4585060	4585074	GATACAGTGCAATAC	+
PA4108	Cyclic di-GMP phosphodiesterase	4591059	4591073	GATACAAAGCGATAC	+
PA4168 (*fpvB*)	siderophore	4663738	4663743	TTTACATTTACATAC	+
PA5100 (*hutU*)	amino-acid degradation	5744860	5744874	TATACTTGTATGTAC	-

Supplementary Table 2. Bacteria strains and plasmids.

Reagent or Resource	Source	Identifier
**Bacterial cells and plasmids**		
E. coli BL21(DE3)	Beijing Genesand Biotech Co.,Ltd	Cat# SEC19
E. coli DH5α	Beijing Genesand Biotech Co.,Ltd	Cat# SCC01
P. aeruginosa PAO1	Prof. Wang' Lab	N/A
P. aeruginosa Δ*bfmR*	This study	
P. aeruginosa Δ*bfmS*	This study	
P. aeruginosa Δ*bfmRS*	This study	
pRK415	Previous study	Song et al., 2021
pEX18-Gm	Previous study	Song et al., 2021
pRG970-Km	Previous study	Song et al., 2021

Supplementary Table 3. Primers used in the work.

Purpose/Name	Sequence (5'-3')
**Protein Expression**	
**pET22b-*bfmS*-f**	CTTTAAGAAGGAGATATACATATGATGAAGCTCGCCGTGC
**pET22b-*bfmS*-r**	TGGTGGTGGTGCTCGAGGGGGTCCAGCTCGATGCGCGCGCA
**pET22b-*bfmR*-f**	CTTTAAGAAGGAGATATACATATGATGGAGCATGTCGATCAC
**pET22b-*bfmR*-r**	TGGTGGTGGTGCTCGAGTGGATGGGCCTCGACCAGTCGCACC
**R185A-f**	GACCAGTTGCTCAACC
**R185A-r**	CGCACTGAGTACCCGTTGC
**R202A-f**	TCCATCGACCTGCTGG
**R202A-r**	CGCGTCGAAGATGTCCGCCTC
**D205A-f**	CTGCTGGTCAGCCGCCTG
**D205A-r**	CGCGATGGAACGGTCGAAG
**S209A-f**	CGCCTGCGCCAACG
**S209A-r**	CGCGACCAGCAGGTCGATGG
**R210A-f**	CTGCGCCAACGCCTCG
**R210A-r**	CGCGCTGACCAGCAGGTCG
**R212A-f**	CAACGCCTCGGCGACG
**R212A-r**	CGCCAGGCGGCTGACCAGC
**T227A-f**	GTGCGCAGCGAGGGCTATG
**T227A-r**	CGCCTTGATGTACTCCGGTTCG
**Gene knockout**	
**pEX18-*bfmS*-upstream-f**	AACGACGGCCAGTGCCAAGCTTCGGATACACTGCTTGCAAGCAC
**pEX18-*bfmS*-upstream-r**	CTCCTTCTTAAAGTTAAACGATGGGCCTCGACCAGTCGCAC
**pEX18-*bfmS*-downstream-f**	GTTTAACTTTAAGAAGGAGGGCGCCCGTACAGAGCGATACA
**pEX18-*bfmS*-downstream-r**	TTCGAGCTCGGTACCCGGGGATATGCTTGCATGATCGATGTCTC
**pEX18-*bfmR*-upstream-f**	AACGACGGCCAGTGCCAAGCTTGTGCACGGCCTGCAAGGCCTGC
**pEX18-*bfmR*-upstream-r**	CTCCTTCTTAAAGTTAAACTGCCTGGCTCCCGTGGCGGTTG
**pEX18-*bfmR*-downstream-f**	GTTTAACTTTAAGAAGGAGTCGCCGTGCCGCGCCCGCGCAG
**pEX18-*bfmR*-downstream-r**	TTCGAGCTCGGTACCCGGGGATTTCGAGATCGTTCCACAGCTTG
**qRT-PCR**	
***oprL*-f**	TGCGATCACCACCTTCTACTTC
***oprL*-r**	CGCTGACCGCTGCCTTTC
***fpvG*-f**	CCCGGTCAGAAGGTTCCATA
***fpvG*-r**	TGGAACATGAGCGGATACCA
***femA*-f**	AACCGAACGCCTATACCGAC
***femA*-r**	TTGTTCTGCTCGGGCTTGAT
***pvdA*-f**	GACCTCAACGACAGCTACCC
***pvdA*-r**	GTGTTGTGGTATTCGCGCAG
***fpvB*-f**	AAGCGCTCGTCCTATTACGG
***fpvB*-r**	TAGCAGTCGTTGTACAGCCC
**Reporter Plasmid**	
**pRG970-P_{bfmRS}-f**	GACTGACCTACCCGGGGATCCTGCCTGGCTCCCGTGGCGGTTG

**pRG970-P*bfmRS*-r**	CTCTAGAAGAAGCTTGGGATCCGGCGACATGCCTTCGTCGAGGC
**pRG970-P*fpvG*-f**	GACTGACCTACCCGGGGATCCGGGTTTCGAGGAAGACCCGACGC
**pRG970-P*fpvG*-r**	CTCTAGAAGAAGCTTGGGATCCGAGGCTAACGGTAGGTTAGGGGTC
**pRG970-P*femA*-f**	GACTGACCTACCCGGGGATCCGGGTTCCTCGTGGTCTCTGC
**pRG970-P*femA*-r**	CTCTAGAAGAAGCTTGGGATCCACCTGCGGCGCACGCTGGGC
**pRG970-P*pvdA*-f**	GACTGACCTACCCGGGGATCCTTCCAGTTCCTCTGGATTGG
**pRG970-P*pvdA*-r**	CTCTAGAAGAAGCTTGGGATCCGAGTTGCCGGCCATCTGCCG
**pRG970-P*fpvB*-f**	CTCTAGAAGAAGCTTGGGATCCCTGCAGTGTCCTGGATGGCG
**pRG970-P*fpvB*-r**	GACTGACCTACCCGGGGATCCTGGCGGCTTACTTCTCTTCG
**pRG970-seqencing-F**	ATTCAGGCTGCGCAACTG
**EMSA**	
**P*fpvG*-f**	GAAACATTTGCTTTCATC
**P*fpvG*-r**	GAGGCTAACGGTAGGTTAGG
**P*femA*-f**	GCGTTGCCATCGACATACCGTGCTCCGCAG
**P*femA*-r**	GAAGCGTGCGTACGGGCATGTACAGGGTTC
**P*pvdA*-f**	CACGCCGCTACGCCCTGTGTTGCCCGTTTC
**P*pvdA*-r**	GGTCAGTACGGTACGCATCCCCATCGATG
**P*fpvB*-f**	CATCGGATTCCTGCGAATAC
**P*fpvB*-r**	GCGTTTCTTGTTTATGTGG
