# Supplementary material for: Molecular mechanism of siderophore regulation by the Pseudomonas aeruginosa BfmRS two-component system in response to osmotic stress
Source: Commun Biol. 2024 Mar 9;7:295. doi: 10.1038/s42003-024-05995-z (PMC10924945; doi:10.1038/s42003-024-05995-z)
Supplement: Supplementary file 2 — Description of Additional Supplementary Files [file 42003_2024_5995_MOESM2_ESM.pdf]

Description of Additional Supplementary Files

File name: Supplementary Data 1
Description: Differentially expressed proteins in bfmRS-mutant compared with WT.

File name: Supplementary Data 2
Description: Differentially expressed proteins in WT treated with high osmolality compared with WT under low osmolality.

File name: Supplementary Data 3
Description: The source data behind the figures in the paper.
