# Supplementary material for: Molecular mechanism of siderophore regulation by the Pseudomonas aeruginosa BfmRS two-component system in response to osmotic stress
Source: Commun Biol. 2024 Mar 9;7:295. doi: 10.1038/s42003-024-05995-z (PMC10924945; doi:10.1038/s42003-024-05995-z)
Supplement: Supplementary file 6 — Reporting Summary [file 42003_2024_5995_MOESM6_ESM.pdf]

nature portfolio

Corresponding author(s): Rui Bao

Last updated by author(s): Feb 7, 2024

Reporting Summary

Nature Portfolio wishes to improve the reproducibility of the work that we publish. This form provides structure for consistency and transparency in reporting. For further information on Nature Portfolio policies, see our Editorial Policies and the Editorial Policy Checklist.

Statistics

For all statistical analyses, confirm that the following items are present in the figure legend, table legend, main text, or Methods section.

n/a	Confirmed	
☐	☒	The exact sample size (*n*) for each experimental group/condition, given as a discrete number and unit of measurement
☐	☒	A statement on whether measurements were taken from distinct samples or whether the same sample was measured repeatedly
☐	☒	The statistical test(s) used AND whether they are one- or two-sided<br>*Only common tests should be described solely by name; describe more complex techniques in the Methods section.*
☒	☐	A description of all covariates tested
☐	☒	A description of any assumptions or corrections, such as tests of normality and adjustment for multiple comparisons
☐	☒	A full description of the statistical parameters including central tendency (e.g. means) or other basic estimates (e.g. regression coefficient) AND variation (e.g. standard deviation) or associated estimates of uncertainty (e.g. confidence intervals)
☐	☒	For null hypothesis testing, the test statistic (e.g. *F*, *t*, *r*) with confidence intervals, effect sizes, degrees of freedom and *P* value noted<br>*Give P values as exact values whenever suitable.*
☒	☐	For Bayesian analysis, information on the choice of priors and Markov chain Monte Carlo settings
☒	☐	For hierarchical and complex designs, identification of the appropriate level for tests and full reporting of outcomes
☒	☐	Estimates of effect sizes (e.g. Cohen's *d*, Pearson's *r*), indicating how they were calculated

Our web collection on statistics for biologists contains articles on many of the points above.

Software and code

Policy information about availability of computer code

Data collection	No software was used
Data analysis	The MS data were analyzed using Spectronaut 17, the data were analyzed using Gaphpad Prism 7.0.

For manuscripts utilizing custom algorithms or software that are central to the research but not yet described in published literature, software must be made available to editors and reviewers. We strongly encourage code deposition in a community repository (e.g. GitHub). See the Nature Portfolio guidelines for submitting code & software for further information.

Data

Policy information about availability of data

All manuscripts must include a data availability statement. This statement should provide the following information, where applicable:

- Accession codes, unique identifiers, or web links for publicly available datasets
- A description of any restrictions on data availability
- For clinical datasets or third party data, please ensure that the statement adheres to our policy

The MS raw files and proteome sequences used in this study have been deposited to the ProteomeXchange Consortium via the iProX partner repository with the dataset identifier PXD046481.

Human research participants

Policy information about studies involving human research participants and Sex and Gender in Research.

Reporting on sex and gender	No such information was collected.
Population characteristics	Our research does not involve such information.
Recruitment	Our research does not involve such information.
Ethics oversight	Our research does not involve such information.

Note that full information on the approval of the study protocol must also be provided in the manuscript.

Field-specific reporting

Please select the one below that is the best fit for your research. If you are not sure, read the appropriate sections before making your selection.

☒ Life sciences ☐ Behavioural & social sciences ☐ Ecological, evolutionary & environmental sciences

For a reference copy of the document with all sections, see nature.com/documents/nr-reporting-summary-flat.pdf

Life sciences study design

All studies must disclose on these points even when the disclosure is negative.

Sample size	Sample sizes were chosen based on past experience for these assessments.
Data exclusions	No data was excluded.
Replication	RT-PCR was repeated three times.
Randomization	The mice were given nondescript numeric identifiers, in a random fashion.
Blinding	All experiments were performed by one researcher at any given point in time. Therefore blinding was not possible.

Reporting for specific materials, systems and methods

We require information from authors about some types of materials, experimental systems and methods used in many studies. Here, indicate whether each material, system or method listed is relevant to your study. If you are not sure if a list item applies to your research, read the appropriate section before selecting a response.

Materials & experimental systems

n/a	Involved in the study	
☒	☐	Antibodies
☒	☐	Eukaryotic cell lines
☒	☐	Palaeontology and archaeology
☐	☒	Animals and other organisms
☒	☐	Clinical data
☒	☐	Dual use research of concern

Methods

n/a	Involved in the study	
☒	☐	ChIP-seq
☒	☐	Flow cytometry
☒	☐	MRI-based neuroimaging

Animals and other research organisms

Policy information about studies involving animals; ARRIVE guidelines recommended for reporting animal research, and Sex and Gender in Research

Laboratory animals	Eight-week-old female C57BL/6 mice
Wild animals	None

Reporting on sex	Eight-week-old female C57BL/6 mice were used in this research, but the experimental results should be applicable to both male and female mice, as this is a universal experimental method for conducting bacterial infection experiments. In the experiment, a total of 250 mice were used, and we did not conduct gender based analysis.
Field-collected samples	The study did not involve samples collected from the field.
Ethics oversight	Eight-week-old female C57BL/6 mice were purchased from Chengdu Dashuo Bioscience Co. Ltd. and housed in experimental animal house of West China School of Public Health, Sichuan University. All animal experiments were reviewed and approved by the Animal Ethics and Safety Review Committee of Sichuan Normal University (Permit Number: 2023LS037) and carried out in compliance with institutional guidelines concerning animal use and care at Sichuan Normal University.

Note that full information on the approval of the study protocol must also be provided in the manuscript.
